# Rate-limiting transport of positively charged arginine residues through the Sec-machinery is integral to the mechanism of protein secretion

**DOI:** 10.7554/eLife.77586

**Published:** 2022-04-29

**Authors:** William J Allen, Robin A Corey, Daniel W Watkins, A Sofia F Oliveira, Kiel Hards, Gregory M Cook, Ian Collinson

**Affiliations:** 1 School of Biochemistry, University of Bristol, University Walk Bristol United Kingdom; 2 School of Chemistry, University of Bristol, University Walk Bristol United Kingdom; 3 Department of Microbiology and Immunology, University of Otago Dunedin New Zealand; University of Kansas Medical Center United States; Goethe University Germany

**Keywords:** protein secretion, membrane transport, SecYEG, SecA, protein translocation, *E. coli*

## Abstract

Transport of proteins across and into membranes is a fundamental biological process with the vast majority being conducted by the ubiquitous Sec machinery. In bacteria, this is usually achieved when the SecY-complex engages the cytosolic ATPase SecA (secretion) or translating ribosomes (insertion). Great strides have been made towards understanding the mechanism of protein translocation. Yet, important questions remain – notably, the nature of the individual steps that constitute transport, and how the proton-motive force (PMF) across the plasma membrane contributes. Here, we apply a recently developed high-resolution protein transport assay to explore these questions. We find that pre-protein transport is limited primarily by the diffusion of arginine residues across the membrane, particularly in the context of bulky hydrophobic sequences. This specific effect of arginine, caused by its positive charge, is mitigated for lysine which can be deprotonated and transported across the membrane in its neutral form. These observations have interesting implications for the mechanism of protein secretion, suggesting a simple mechanism through which the PMF can aid transport by enabling a 'proton ratchet', wherein re-protonation of exiting lysine residues prevents channel re-entry, biasing transport in the outward direction.

## Introduction

Secretion of proteins synthesised in the cytosol is governed by cleavable N-terminal signal sequences (SS), necessary and sufficient for targeting (Blobel and Dobberstein, 1975), possibly augmented by information in the mature protein such as a lowered propensity to fold and exposed hydrophobic patches (Chatzi et al., 2017). Secretory and membrane proteins are recognised by factors for delivery, usually in an unfolded state, to bespoke protein translocation machineries (translocons) for carriage across or into cellular membranes. Amongst these, and responsible for the majority both of protein secretion and membrane protein insertion, is the universally conserved Sec system: at its core the heterotrimer SecYEG/β in plasma membrane of bacteria and Archaea or Sec61αβγ in the endoplasmic reticulum of eukaryotes. The Sec system transports unfolded proteins, either immediately as they emerge from the ribosome (co-translationally) or following their release (post-translationally; Arkowitz et al., 1993).

**Figure 1.**
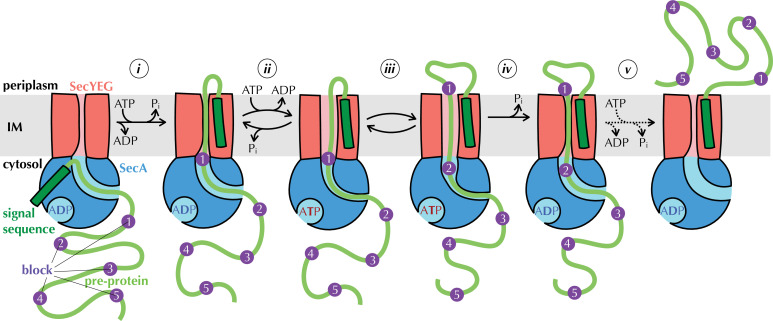
The Brownian ratchet model. Mechanism of pre-protein transport, based on Allen et al., 2016. Cycles of ATP binding and hydrolysis by SecA (blue) allow blocks (purple) in the pre-protein substrate (green) to diffuse outwards through SecYEG (red) one at a time, but backsliding is prevented, leading to directional transport. See text for more detail.

In bacteria, membrane protein insertion is generally co-translational, whereas almost all secretion is post-translational, mediated by the cytosolic ATPase SecA (Lill et al., 1990). Prior to passage across the membrane, pre-proteins with a SS are recognised by SecA, either directly or with the assistance of chaperones such as SecB, and brought to SecYEG in the membrane (Figure 1; Hartl et al., 1990; Kumamoto and Beckwith, 1983; Oliver and Beckwith, 1982). The SS unlocks the translocon and initiates transport by inserting as a hairpin through the channel (step *i* in Figure 1; Corey et al., 2016b; Ma et al., 2019), using ATP hydrolysis by SecA as an energy source (Economou and Wickner, 1994; Fessl et al., 2018; Lill et al., 1990). Transport of the rest of the of the polypeptide substrate proceeds stepwise (steps *ii-v*, elaborated below), with cycles of ATP turnover (Brundage et al., 1990), aided in vivo by the electrochemical gradient of protons across the membrane – the proton-motive force (PMF; positive and acidic outside; Schiebel et al., 1991). Once the pre-protein has crossed the membrane, the SS is cleaved off by signal peptidase and the transported protein released (Josefsson and Randall, 1981); this release step appears to require additional factors – most likely the periplasmic chaperone PpiD (Antonoaea et al., 2008; Fürst et al., 2018) – as it is very slow when measured in vitro using ex vivo or purified and reconstituted components (Allen et al., 2020; Mao et al., 2020).

**Figure 1—figure supplement 1.**
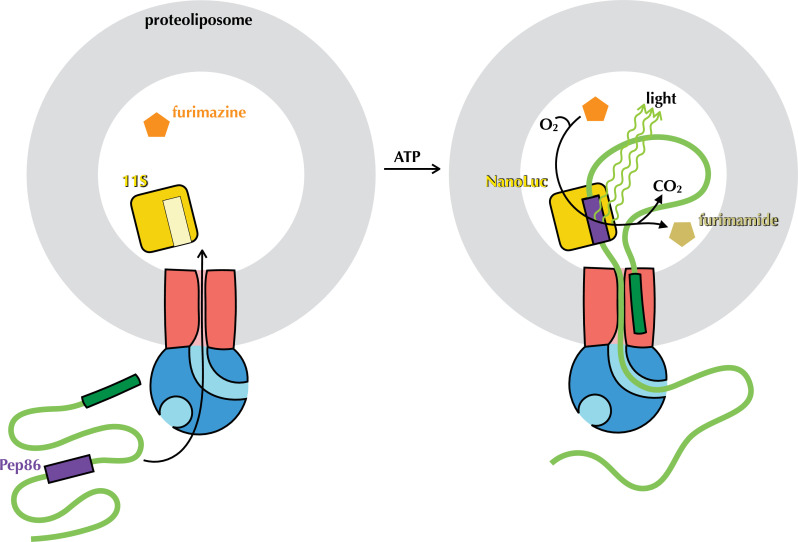
The NanoLuc pre-protein transport assay. Proteoliposomes (grey) with encapsulated 11S (yellow) and SecYEG in the membrane (red) are supplied with SecA (blue), pre-protein (green) containing pep86 (purple) and furimazine (orange). Upon addition of ATP, pre-protein is imported into the PLs, allowing pep86 to complement 11S, poducing NanoLuc. This gives a luminescence signal proportional to the amount of NanoLuc formed, for as long as furimazine and O_2_ remain in large excess over furimamide and CO_2_.

Results from our laboratory and elsewhere, using the model *Escherichia coli* Sec system, have shown how ATP turnover could be coupled to directional pre-protein movement (Allen et al., 2016; Catipovic et al., 2019; Catipovic and Rapoport, 2020; Corey et al., 2019). Adding a non-hydrolysable ATP analogue to the system, which emulates the effect of nucleotide exchange (step *ii* in Figure 1) has two major observable effects: it widens the channel through SecYEG (Allen et al., 2016) while tightening the clamp around pre-protein in SecA (Ahdash et al., 2019; Catipovic et al., 2019). Nucleotide exchange itself is promoted by perturbation of the two-helix finger (2HF; Allen et al., 2016; Zimmer et al., 2008) of SecA caused by the presence of pre-protein at the entrance to the channel through SecYEG (Allen et al., 2016). Together, these observations led us to propose a model in which pre-protein moves through the channel primarily by diffusion (Allen et al., 2016). Sequences that cannot diffuse across the membrane in the ADP-bound state (blocks, purple in Figure 1) trigger nucleotide exchange (step *ii*), opening the SecYEG channel to allow them to slide through (step *iii*) and simultaneously clamping SecA shut to prevent them slipping backwards. ATP hydrolysis resets the channel (step *iv*), trapping block sequences that have diffused across on the outside of the membrane, thus providing directionality. This process (steps *ii-iv*) is repeated for each remaining block (step *v*) until the entire pre-protein has crossed the membrane.

A major prediction of the above model is that not every ATP turnover will give rise to a transport event, as step *ii* is reversible. Recent high-resolution protein transport data, collected using a new assay based on split NanoLuc luciferase (Dixon et al., 2016; Pereira et al., 2019), lent support to this notion. In this assay, the large fragment of NanoLuc (11S) is encapsulated within proteoliposomes (PLs) or inverted membane vesicles (IMVs) containing SecYEG, while a high-affinity complementary small fragment (pep86) is incorporated into the pre-protein (Figure 1—figure supplement 1). As soon as pep86 enters the vesicle it combines with 11S, producing a luminescence signal (Figure 1—figure supplement 1; Allen et al., 2020; Pereira et al., 2019). Using detailed kinetic modelling, we showed that transport occurs in about five steps for the model 161 amino acid pre-protein pSpy, and that each step requires many ATP turnovers to resolve (an estimated 120 for pSpy in vitro, see Allen et al., 2020). However, we were unable to show exactly what these steps – presumably the blocks in the above model – physically correspond to. It also remains unclear how the PMF contributes.

To answer these questions, we have now generated a range of different pSpy variants with different physical and chemical properties. Employing the NanoLuc assay to measure their transport both in the presence and absence of proton-motive force (PMF), we show that transporting positively charged residues across the membrane is the slowest step of transport, with bulkier residues also increasing secretion time. This strong barrier to positive charges is partially overcome by deprotonating lysines at the cytosolic face of the membrane; for this reason, arginines, which have a much higher p*K*_a_ than lysine, are by far the hardest amino acid to transport. Surprisingly, however, charged residues seem unaffected by the electrical component of PMF (∆ψ) in our experimental setup – even though electrophoresis ought to be favourable, given that deprotonation of lysines confers a net negative charge to the polypeptide as it passes though the channel towards the positive exterior.

We also find that removing all arginine residues from the pre-protein hugely increases the number of kinetic transport steps, despite speeding up transport overall. Variable step size is characteristic of diffusion-based model such as the one presented above (Figure 1). For a power stroke mechanism, on the other hand, for example where an ATP-dependent piston movement of SecA pushes stretches of polypeptide across the membrane (Catipovic et al., 2019; Catipovic and Rapoport, 2020) step size depends on SecA, and should thus be invariant. Taken together, our results provide important new mechanistic insights of the mechanism of protein secretion; both the insights and the experimental approaches are potentially applicable to other machineries that transport unfolded proteins.

## Results

### The chemical and physical properties of pre-protein affect its transport characteristics

**Figure 2.**
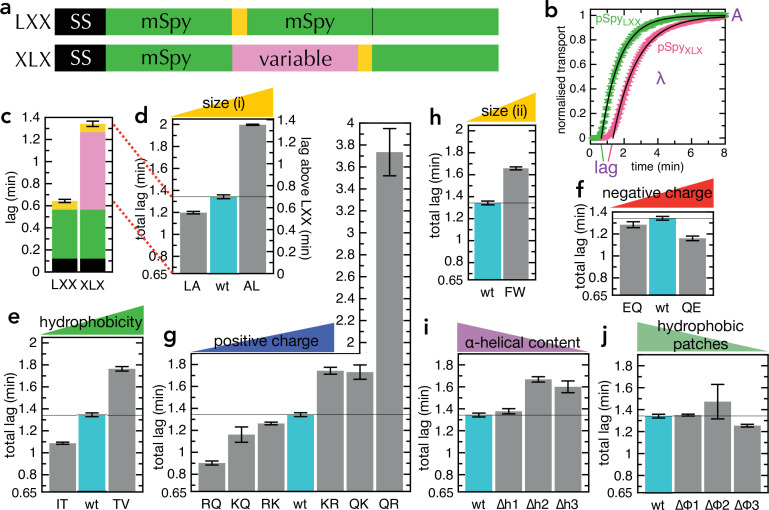
Transport of pSpy_XLX_ variants. (**a**) Schematic of pSpy_LXX_ (LXX) and pSpy_XLX_ (XLX). Transport occurs from the N- to C-terminus (left to right), and a luminescence signal appears as soon as pep86 (yellow) enters the lumen of the PL. In these constructs 'X' refers to mSpy with no pep86, and 'L' refers to mSpy followed by a functional (Light) pep86. (**b**) Transport traces of pSpy_LXX_ (green) and pSpy_XLX_ (pink), normalised to give an amplitude (A; signal when the transport reaction reaches completion) of 1, and fitted to the simple lag +single exponential model (Allen et al., 2020). In this model, lag is the minimum time required for pre-protein transport, and corresponds to the sum of time constants for all transport steps (equal to 1*/k*, where *k* is the rate constant for that step). λ is a complicated variable, incorporating the transport rates but also the probability of transport pausing or failure and resetting (see Allen et al., 2020 for more details). Data are the average and SEM from three (pSpy_LXX_) or 11 (pSpy_XLX_) experimental replicates. (**c**) Lag (taken from panel **b**) for pSpy_LXX_ and pSpy_XLX_. (**d-j**) Lags for a range of pSpy_XLX_ variants, where the central mSpy has its chemical and physical properties varied (see text for details; wt (wild type) is native Spy). In each case, the y-axis starts at the transport time for pSpy_LXX_, so the visible part of the bar corresponds to the transport time only of the variable region. Data show the average and SEM from three experimental replicates. Almost all the variants are statistically significant from wt; a table of p-values is included in Supplementary file 3a. Figure 2—source data 1.Raw data for Figure 2 and Figure 3.

We started by investigating which physical properties of a pre-protein determine how fast it is transported through SecYEG, by systematically varying the amino acid sequence of the model Sec substrate pSpy. It quickly became apparent that altering pSpy affects its solubility in transport buffer, potentially along with other properties such as affinity for SecA, and the rates of initiation and termination. We therefore created constructs consisting of the pSpy SS followed by three tandem mature (m)Spys, with a pep86 sequence after the second (pSpy_XLX_; XLX in Figure 2a) – and altered only the central one. The two flanking native Spy sequences ensure that the beginning and end of transport are always the same, and prevent the less stable Spy variants from precipitating upon dilution out of urea. As a control, we created the same construct but with pep86 after the first mSpy (pSpy_LXX_, LXX in Figure 2a). The difference in transport time between these two proteins (measured from the lag before transport signal appears, see Figure 2b and Allen et al., 2020) corresponds exactly to the time it takes to transport the central mSpy (pink in Figure 2a and c). The lags for these match perfectly with those for the pSpy_4x_ series used previously (four tandem Spys; Allen et al., 2020; Figure 2—figure supplement 1a-b), confirming that lag is indeed a true and sensitive measure of transport time.

**Figure 2—figure supplement 1.**
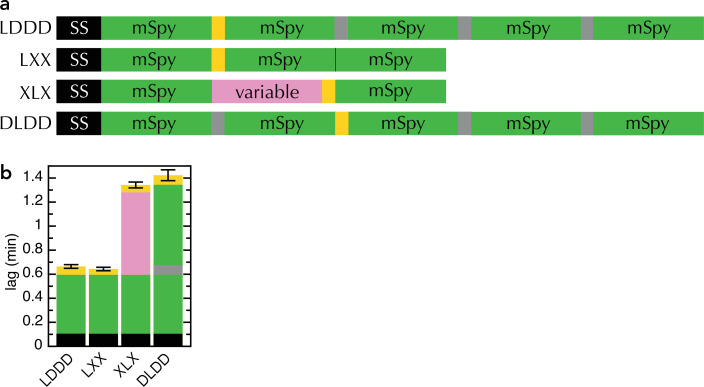
Comparison of new and old transport substrates. (**a**) Schematic of pSpy_LXX_, pSpy_XLX_, pSpy_LDDD_ and pSpy_DLDD_, as in Figure 1a, where 'D' refers to mSpy followed by an inactive (Dark) pep86 (Allen et al., 2020). (**b**) Transport lags for the pSpy variants in panel (**a**). Those for pSpy_LXX_ and pSpy_XLX_ are the same as in Figure 2c (i.e. average and SEM of 3 and 11 replicates, respectively), while those for pSpy_LDDD_ and pSpy_DLDD_ are taken from Allen et al., 2020 (i.e average and SEM of 8 repeats).

**Figure 2—figure supplement 2.**
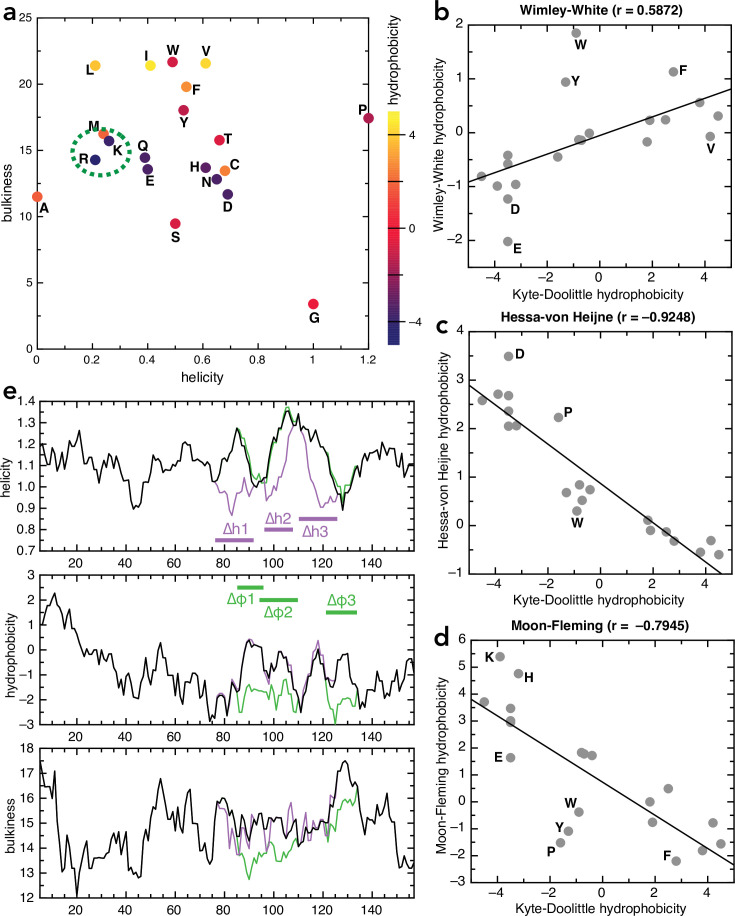
Amino acid and sequence properties. (**a**) Plot of all 20 naturally encoded amino acid by hydrophobicity (scale from Kyte and Doolittle, 1982), helical propensity (scale from Deléage and Roux, 1987) and bulkiness (scale from Zimmerman et al., 1968). (**b–d**) Comparison of different hydrophobicity scales, determined empirically for membrane proteins, with the Kyte-Doolittle scale: (**b**) Wimley-White (Wimley and White, 1996), (**c**) Hessa-von Heijne (Hessa et al., 2005), and (**d**) Moon-Fleming (Moon and Fleming, 2011). Outliers are marked on the plots; in general the scales correlate well, with differences mainly in the aromatic residues. (**e**) Plots of helicity (top), hydrophobicity (middle), and bulkiness (bottom) of native pSpy (black) and the helicity (purple) and hydrophobicity (green) variants, as a function of sequence position, averaged over a window of nine amino acids. Calculations performed using ProtScale on the Expasy portal, using the same scales as in panel (**a**).

Our first set of pSpy_XLX_ variants were created by replacing 6–8 of one type of residue with another, distributed as evenly as possible through mSpy (all sequences shown in Supplementary file 1). We chose four general amino acid properties: size (L→A, A→L; Figure 2d), hydrophobicity (T→V, I→T; Figure 2e), negative charge (Q→E, E→Q; Figure 2f), and positive charge (R→Q, Q→R, K→Q, Q→K, K→R, R→K; Figure 2g). In the case of positive charge, we looked both at lysine – which can in some environments be deprotonated at physiological pH (Isom et al., 2011) – and arginine, which is generally considered to always retain its positive charge (Harms et al., 2011 see also below). We find that transport is slower (longer lag than native pSpy_XLX_ above that of pSpy_LXX_, 0.65 min) when more bulky, hydrophobic and positively charged residues are present, while negative charges have limited effect. By far, the strongest effect comes from changing the number of arginines: removing all eight (RQ in Figure 2g) reduces the transport time threefold (from 0.64 min to 0.26 min), while adding a further eight (QR in Figure 2g) slows transport over fourfold (to 3.1 min).

Leucine is more hydrophobic than alanine, while threonine is somewhat smaller than isoleucine or valine (Figure 2—figure supplement 2a), so these experiments do not distinguish particularly effectively between bulkiness and hydrophobicity. We therefore designed an additional variant in which all five phenylalanine residues were replaced with tryptophan (F→W; Figure 2h). This increases bulkiness while decreasing hydrophobicity according to most hydrophobicity scales (Figure 2—figure supplement 2a-d), in theory allowing the two effects to be disentangled. The results reveal that the F→W substitutions slow transport, which suggests that residue size is a more important factor than hydrophobicity. It should be noted, however, that tryptophan is more hydrophobic than phenylalanine according to the Wimley-White scale (Wimley and White, 1996); hence, this substitution is not an absolute confirmation of the importance of size over hydrophobicity.

We have previously shown that the ATP hydrolytic cycle of SecA can influence the formation of secondary structure (primarily α-helix) within the translocating pre-protein (Corey et al., 2019), promoting its unfolding at the cytosolic entrance to SecYEG and formation on the periplasmic side. To explore the effect of this on transport kinetics, we designed pSpy_XLX_ variants in which the helical propensity of one, two or three regions was reduced, without affecting the hydrophobic character of that region (∆h1, ∆h2 and ∆h3; Figure 2i; Figure 2—figure supplement 2e, purple). A converse set of mutations, in which the hydrophobic character is reduced without altering the helical propensity, was also generated (∆φ1, ∆φ2 and ∆φ3; Figure 2j; Figure 2—figure supplement 2e, green). The transport parameters with these variants show that if sufficient helical content is removed it somewhat slows transport (Figure 2i), supporting the notion that helix formation is part of the mechanism of transport (Corey et al., 2019). Removing hydrophobic patches, meanwhile, has marginal if any effect on transport rate (Figure 2j); taken together with the other results above, this suggests residue size is a bigger factor than hydrophobicity in determining transport rate.

### Specific measurement of the transport steps for the pSpy_XLX_ variants

The lag is a useful measure of overall transport time, but it cannot distinguish between a large number of fast steps and a small number of slow steps. For this, we employed a numerical model of transport, derived previously and implemented in Berkeley Madonna (Figure 3—figure supplement 1a; Allen et al., 2020 see Materials and methods). In this model, binding of pre-protein to the Sec system (with on and off rates *k*_on_ and *k*_off_) starts at equilibrium. Addition of ATP starts the reaction, allowing initiation (*k*_init_, equal to *k*_step_ and corresponding to step *i* in Figure 1) followed by n kinetic transport steps (each one equivalent to steps *ii-iv* in Figure 1) with the rate *k*_step_. The final step gives rise to a luminescent product, as NanoLuc formation is essentially instant on the time scale of transport (Allen et al., 2020). Two additional rate constants are required to describe the data fully: *k*_fail_, the rate at which translocating pre-protein dissociates and transport can be restarted; and *k*_block_, where the pre-protein becomes permanently trapped in the channel, preventing further transport at that site. A final parameter, 'brightness', simply represents the amount of luminescent signal per NanoLuc, normalised to give a maximum signal of 1.

**Figure 3.**
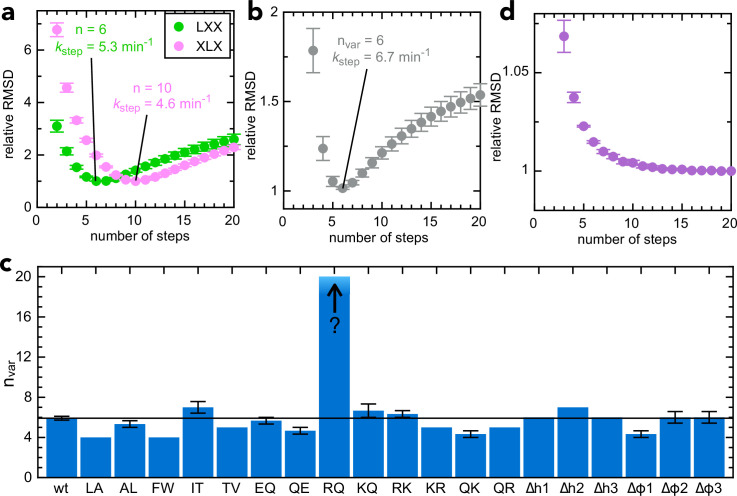
Determining step size for the variable mSpy regions. (**a**) RMSD (normalised to its lowest value) for fits to the experimental transport data to the original transport model (see Figure 3—figure supplement 1a and Allen et al., 2020), over a range of values for n (number of steps), using Berkeley Madonna. Error bars are the SEM from 4 (pSpy_LXX_) or 12 (pSpy_XLX_) replicates. Values for other fixed parameters are *k*_block_ = 0.31 min^–1^ (determined previously; Allen et al., 2020, *k*_on_ = 0.96 µM^–1^.min^–1^ see Figure 3—figure supplement 2a) and k_off_ = 0.085 min^–1^ (see Figure 3—figure supplement 2b). Best fits are to 6 steps for pSpy_LXX_ (green; *k*_step_ = 5.31 ± 0.05 min^–1^) and 10 steps for pSpy_XLX_ (pink; *k*_step_ = 4.57 ± 0.15 min^–1^). (**b**) RMSD (normalised to its lowest value) for fits of experimental transport data for pSpy_XLX_ to the model in Figure 3—figure supplement 1b, over a range of values for n_var_. All parameters other than *k*_step,var_, *k*_fail,var_ and brightness are fixed to the same values as in panel (**a**). The best fit is to n_var_ = 6, *k*_step,var_ = 6.74 ± 0.24 min^–1^. (**c**) Best fit n_var_ for each of the pSpy_XLX_ variants in Figure 2d–j, calculated as in panel (**b**), but with brightness adjusted for the values in Figure 3—figure supplement 3 (see text for details), and *k*_block,var_ allowed to float. Errors are the SEM from 12 (wt) or 3 (all others) replicates. (**d**) Normalised RMSD as a function of n_var_ for pSpy_XLX_^R→Q^ (all parameters as in panel **c**).

Just as described previously (Allen et al., 2020), best fits to the experimental data were calculated using the Curve Fit function of Berkeley Madonna over a range of values for n, allowing *k*_step_, *k*_fail_ and brightness to vary but fixing the other rate constants to previously determined values. The best fit is taken as the one with the lowest root mean square deviation (RMSD) between the model and the data, normalised to 1 to allow comparison between different data sets. Using this analysis, we find that transport of pSpy_LXX_ is best described by 6 steps with an average *k*_step_ of 5.30 min^–1^ (green in Figure 3a), while transport of pSpy_XLX_ proceeds in 10 steps with *k*_step_ = 4.42 min^–1^ (pink in Figure 3a).

**Figure 3—figure supplement 1.**
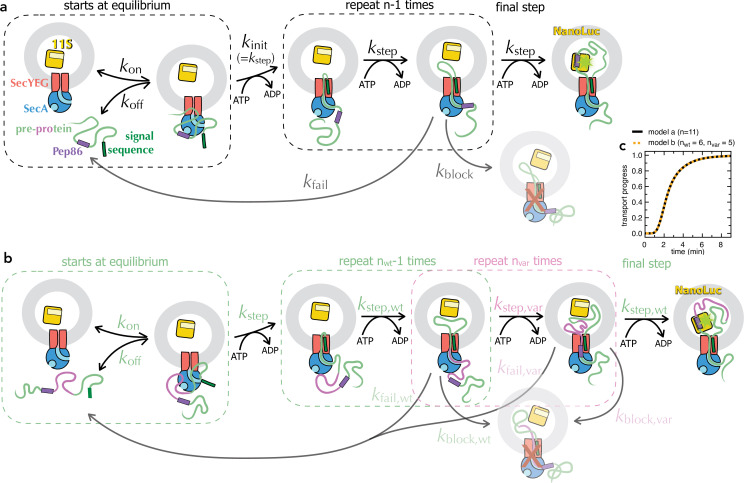
Schematic representations of numerical models. (**a**) Minimal model for transport of pre-protein through the Sec system (Allen et al., 2020). Before the addition of ATP, pre-protein associates with SecYEG and SecA, with an affinity dependent on both the SS and mature domain of the preprotein (K_d_ ~0.6 µM for native proSpy, Allen et al., 2020). Starting the reaction with ATP allows transport to initiate (*k*_init_, equal to *k*_step_), followed by a number (**n**) of steps, each with the rate *k*_step_. As soon as Pep86 enters the PL it binds to 11S, producing the luminescent signal. Because there are multiple sequential steps before NanoLuc formation, the transport signal begins to appear after a characteristic lag, equal to *k*_init_ + n × *k*_step_ (Allen et al., 2020). Not every transport event that initiates goes on to produce a functional NanoLuc. At any point, transport can fail: either dissociating and requiring reinitiation (*k*_fail_); or permanently, blocking the channel and preventing further transport (*k*_block_). In the case of proSpy, each SecYEG appears only to transport a single pre-protein – so the total signal amplitude depends on the number of SecYEG import sites, and the relative rates of transport and *k*_block_ (Allen et al., 2020). Thus, simple analysis of the NanoLuc traces can provide estimates for the rate of transport and its success rate. (**b**) Extended transport model, separating out transport of the second mSpy of pSpy_XLX_ (pink) from all the other steps (binding, initiation and transport of a single mSpy and Pep86; green). For fitting, all parameters in green are fixed at the values determined from pSpy_LXX_. Brightness is fixed to relative to the wild type pSpy_XLX_ according to the values determined in solution (Figure 3—figure supplement 3a-f, turquoise bars). The three remaining rate constants (*k*_block,var_, *k*_step,var_ and *k*_fail,var_) are allowed to float, and n_var_ is varied systematically. This model is defined for Berkeley Madonna in Supplementary file 2. As long as the wt and var rate constants are all kept the same, model (**a**) is identical to model (**b**) if n in **a** is equal to n_wt_ +n_var_ in **b** (see panel **c**). (**c**) Simulated transport curves using the original model (panel **a**) with n = 11 (black solid line), or the split model (panel **b**) with n_wt_ = 6 and n_var_ = 5 (orange dotted line), and all other parameters kept the same. The fact that these overlay perfectly confirms that the no errors were introduced in extending the model.

**Figure 3—figure supplement 2.**
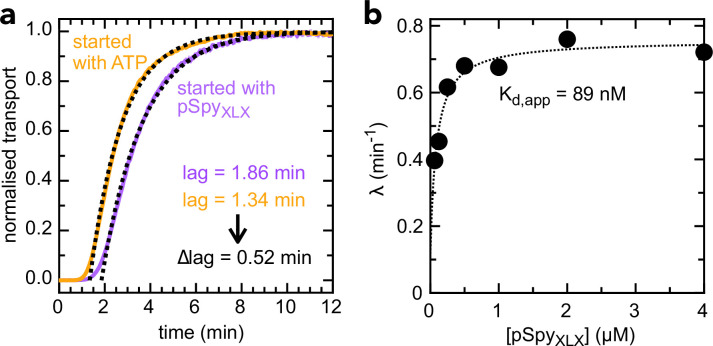
Determination of *k*_on_ and *k*_off_ values for pSpy_XLX_. (**a**) Transport of 2 µM pSpy_XLX_ initiated either by the addition of ATP (orange line) or pSpy_XLX_ (purple line), along with fits (black dotted lines). The difference in lag (0.52 min) corresponds to the time it takes for pSpy_XLX_ to associate with SecYEG-SecA, i.e. *k*_on_^–1^ (see Allen et al., 2020 for details). This gives *k*_on_ = 0.96 µM^–1^.min^–1^, very similar to the value previously determined for native pSpy (*k*_on_ = 1.25 µM^–1^.min^–1^ [Allen et al., 2020]): not surprising, given that this interaction is driven primarily by signal sequence and early mature domain which are identical in both proteins. (**b**) λ (the exponential component of the fit, see Allen et al., 2020) as a function of pSpy_XLX_ concentration. Fitting to a simple weak binding equation (λ = λ_0_ + [pSpy_XLX_] ÷ ([pSpy_XLX_] + K_d_)) gives a good approximation of K_d_ for the pSpy_XLX_–Sec interaction, as it reflects the starting equilibrium of the binding step (see Figure 3—figure supplement 1a and Allen et al., 2020). From the best fit value (K_d_ = 0.089 µM) and *k*_on_ (0.96 µM^–1^.min^–1^, panel a), we estimate *k*_off_ = 0.085 min^–1^ (from the definition K_d_ = *k*_off_ ÷ *k*_on_). Figure 3—figure supplement 2—source data 1.Raw data for Figure 3—figure supplement 2a. Figure 3—figure supplement 2—source data 2.Raw data for Figure 3—figure supplement 2b.

**Figure 3—figure supplement 3.**
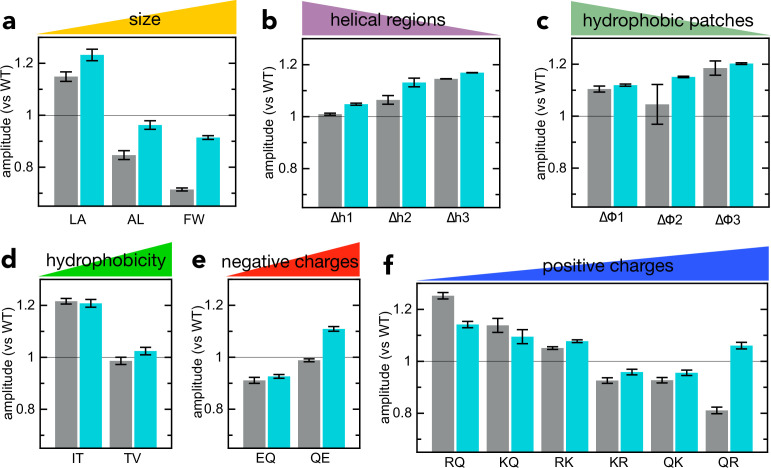
Comparison of signal amplitudes for NanoLuc transport vs solution binding. (**a–f**) Grey bars: amplitudes for the import of the various pSpy_XLX_ variants (same primary data as in Figure 2), normalised to the amplitude of native pSpy_XLX_ (measured on the same plate in the same experiment). Turquoise bars: amplitude for binding of 2 µM of each pSpy_XLX_ variant to 20 pM 11S free in solution. In each case, the signal from 20 pM 11S with only buffer is subtracted, and the amplitude normalised to the value of native pSpy_XLX_ (both measured on the same plate in the same experiment). Error bars are the SEM from four experimental replicates (eight for native pSpy_XLX_ and pSpy_LXX_). With a few exceptions, transport and binding amplitude correspond well, indicating that differences in amplitude are due to differences in the luminescence of NanoLuc (brightness in the model in Figure 3—figure supplement 1). Figure 3—figure supplement 3—source data 1.Raw data for Figure 3—figure supplement 3.

To extract n specifically for transport of the second mSpy of pSpy_XLX_ (Figure 2a, pink; the variable one in the above constructs; n_var_), we split the Berkeley Madonna mathematical model into two parts (Figure 3—figure supplement 1b) – transport of the 'native' section and the 'variable' section. All parameters for transport of the native mSpy (including initiation and pep86 transport) were fixed to the best fit for pSpy_LXX_ (n = 6, *k*_step_ = 5.30 min^–1^), while *k*_step_ for the variant (*k*_step,var_) was allowed to float and the best fit determined over a range of values for n_var_. This gives a best fit of n = 6 for pSpy_XLX_ (*k*_step_ = 6.74 ± 0.24 min^–1^; Figure 3b), in reasonable agreement with the fit to the simpler model (Figure 3a).

We next used this model to extract n_var_ for each of the 19 pSpy_XLX_ variants. The amplitudes of each variant (i.e. maximum signal, normalised to that of native pSpy_XLX_ run in parallel) differ somewhat from sequence to sequence (Figure 3—figure supplement 3, grey bars) – due either to differences in the signal produced by each NanoLuc ('brightness' in the Berkeley Madonna model) or in the probability that the sequences become trapped within the channel (*k*_block_). To distinguish these possibilities, we measured the NanoLuc signal of each variant in solution, under saturating conditions (Figure 3—figure supplement 3, turquoise bars). The results suggest that in most but not all cases, the variance is down to small differences in NanoLuc brightness (compare grey and turquoise bars in Figure 3—figure supplement 3). To account for this in the fitting, we fixed brightness for each variant based on its measured value relative to native pSpy_XLX_, then allowed *k*_block,var_ to float, in addition to *k*_step,var_ and k_fail,var_.

The best fit number of steps for each variant is shown in Figure 3c (full fitting results are in Supplementary file 3b). In all but one case, n falls between 4 and 7 (i.e. very similar to the native sequence, n = 6). Strikingly, however, it is not possible to determine a number of steps for pSpy_XLX_^R→Q^: the RMSD continues to go down then platueas as n_var_ increases (at least up to 20; Figure 3d). We conclude that in the vast majority of cases transport time is dominated by a small number of slow steps; removal of arginines by mutagenesis eliminates these, revealing a large number of steps that are otherwise too fast to measure.

To summarise so far: arginines, which have a fixed positive charge, have profound effects on the process of transport; that is, in respect of both the rate and the average number of steps required to transport a given polypeptide across the membrane.

### Lysine is deprotonated to facilitate its passage through SecYEG

**Figure 4.**
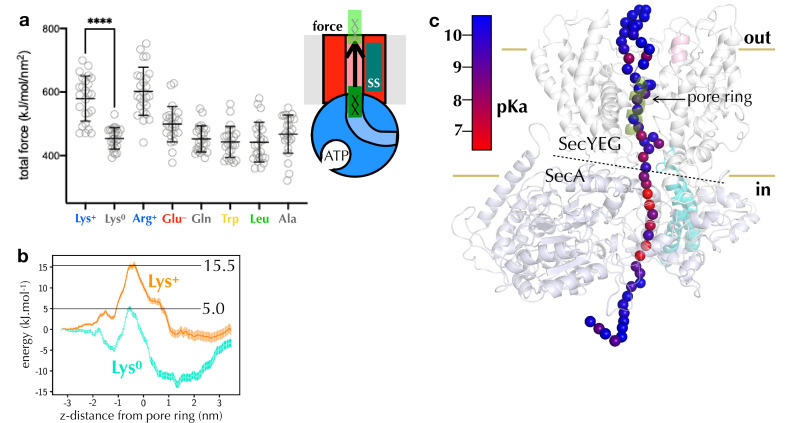
Computational analysis of pre-protein engaged in SecYEG. (**a**) Pulling forces for regions of polypeptide containing different residues of interest to pass the SecY pore, as determined using steered MD. Plotted are the integrated forces along the pull coordinated, with 24 repeats for each residue. The mean and standard deviations are shown. (**b**) Potential of mean force pathways for a short region of peptide with either protonated (Lys^+^) or deprotonated (Lys^0^) present passing through the SecY pore. These systems were built using the Martini force field. (**c**) p*K*_a_ scanning data for lysine residues at different positions along the substrate. Alpha-carbon positions of the substrate are shown as spheres, and coloured according to their calculated p*K*_a_. Figure 4—source data 1.Raw data for panel a. Figure 4—source data 2.Raw data for panel b.

It has previously been shown that SecYEG is strongly selective against positively charged ions or residues, but more permeable to anions (Dalal and Duong, 2009; Nouwen et al., 2009; Schiebel and Wickner, 1992). However, the specific effect of arginine has not hitherto been described. To explore this further, we carried out constant velocity steered molecular dynamics (SMD), to explore how easy it is for different side chains to pass through the channel. Here, a 14-residue stretch of polypeptide, based on the PDB structure 5EUL (Li et al., 2016), containing a residue of interest is pulled through the SecY pore (see Materials and methods), and the total force experienced along this pull coordinate is recorded (Figure 4a). We found that the total force required to move positive charges across the membrane is significantly higher than any other residue type, but with no difference between protonated lysine and arginine (Figure 4a). The effect is specifically due to the positive charge: uncharged (deprotonated) lysine transports just as easily as other uncharged residues (e.g. Lys^0^ vs Leu in Figure 4a). We quantified this effect using potential of mean force calculations with the coarse-grained Martini force field (Marrink et al., 2007; Monticelli et al., 2008), which predicts a difference in energy barrier of ca. 10 kJ mol^–1^ between charged and uncharged lysine (Figure 4b).

The fact that lysine is transported more easily than arginine in vitro but not in silico would be explained if lysine loses its positive charge before traversing the channel. The p*K*_a_ of lysine in solution is ~10, but it is readily deprotonated in hydrophobic environments (Isom et al., 2011), whereas the delocalised positive charge of arginine’s guanidinium group (p*K*_a_ ~13.8 [Fitch et al., 2015]) is much harder to remove (Harms et al., 2011). Additionally, the ∆pH component of the PMF would assist with the deprotonation of lysines at the entrance to SecY, and with their rapid reprotonation in the periplasm.

To explore this possibility, we carried out an in silico p*K*_a_ analysis, in which a long stretch of native pre-protein substrate is threaded through the SecA-SecYEG channel (Zimmer et al., 2008), and relaxed over 1 µs of atomistic MD simulation (see Materials and methods). For multiple simulation snapshots, residues along the pre-protein were then mutated to lysine, relaxed with MD, and the p*K*_a_ recorded using the propKa31 program (Søndergaard et al., 2011). The analysis reveals a clear region of p*K*_a_ perturbation at the SecYEG-SecA interface (Figure 4c), in line with our predictions. Aside from their p*K*_a_s, lysines and arginines are very similar in terms of their physical and chemical properties (circled in Figure 2—figure supplement 2a). The specific deprotonation of lysines therefore seems to be the only plausible explanation for the huge differential effect of arginine relative to lysine on transport time.

### Arginine transport exerts a selection pressure on secreted proteins

**Figure 5.**
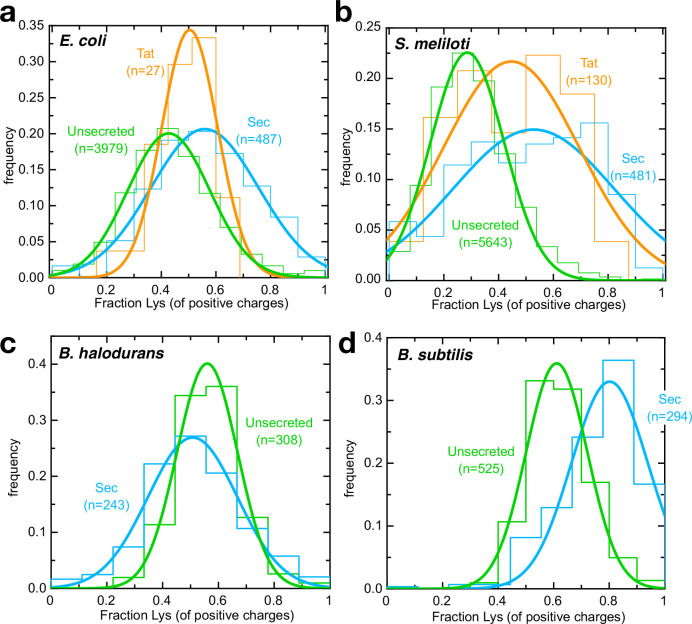
Arginines are selected against for secretion by neutrophiles. (**a–d**) Histograms (fine lines) showing fraction of positive residues that are lysine in cytosolic proteins (green), Sec substrates (blue) and Tat substrates (orange; panels **a,b** only), for (**a**) *E. coli,* (**b**) *S. meliloti*, (**c**) *B. halodurans* and (**d**) *B. subtilis*. Best fit single Gaussians are also shown (thick lines) for clarity. All differences are statistically significant except for *E. coli* Tat substrates; p-values are shown in Supplementary file 3c. Figure 5—source data 1.Binned Lys/Arg ratios for Figure 5.

If transport of arginines is rate limiting for secretion in vivo, one might expect Sec substrates to experience an evolutionary selection pressure to eliminate arginines, where possible – most likely with lysine, the only other positively changed amino acid at neutral pH. To investigate this, we compared the Lys/Arg composition of the mature domain of all known *E. coli* Sec substrates with those that remain in the cytosol. As predicted, secreted proteins have a strong preference for lysine over arginine relative to those in the cytosol (Figure 5a). We also looked at pre-proteins that are exported, but by the Tat system – that is independently of Sec – and found their Lys/Arg ratio appears to fall somewhere between Sec substrates and unsecreted proteins (Figure 5a). This confirms that the Lys/Arg effect is at least in part a product of transport pathway, rather than the extra-cytosolic environment.

Because *E. coli* only uses Tat to export a very small number of proteins, we carried out the same analysis on a model organism with a large number of annotated Tat substrates (*Sinorhizobium meliloti*; Pickering et al., 2012; Figure 5b). The results both confirm the *E. coli* observations and show that they hold true across different classes of Gram-negative bacteria. The small difference in Lys/Arg ratio for Tat vs unsecreted proteins may be relevant to the mechanism of folded protein export, although it could equally reflect differences in the cytosolic vs periplasmic environment.

To explore whether this effect is related to ∆pH specifically, we carried out the same analysis on an alkalophilic organism with an inverted ∆pH (acid_in_/alkaline_out_), *Bacillus halodurans* (Takami et al., 2000). Consistent with ∆pH being involved in transport, lysine is no longer favoured over arginine for secreted proteins – indeed, the reverse appears to be true (Figure 5c). Meanwhile, the related bacterium *Bacillus subtilis*, which grows in neutral environments (alkaline_in_/acid_out_), shows the expected increase in preference for lysine for secreted substrates (Figure 5d). The fact that this trend holds true in three very distantly related bacteria suggests it could be a general feature of secreted proteins.

### Proton motive force speeds up transport primarily through ∆ψ

The question of how the PMF stimulates SecA-mediated pre-protein transport has been open for decades (Brundage et al., 1990): while it is intuitive to imagine negatively charged residues crossing the membrane electrophoretically with the aid of ∆ψ, the same effect would equally prevent transport of positive charges. Indeed, a strong electrophoretic pulling force has been observed for negatively charged residues at the SecY channel entrance during co-translational membrane protein insertion, but no equivalent slowing of positively charged residues (Ismail et al., 2015). Deprotonation of lysines at the cytosolic face of SecYEG could neatly circumvent this problem, by imbuing essentially all pre-proteins with a net negative charge as they cross the membrane. To investigate this possibility, we therefore set out to measure the effect of PMF on transport.

**Figure 6.**
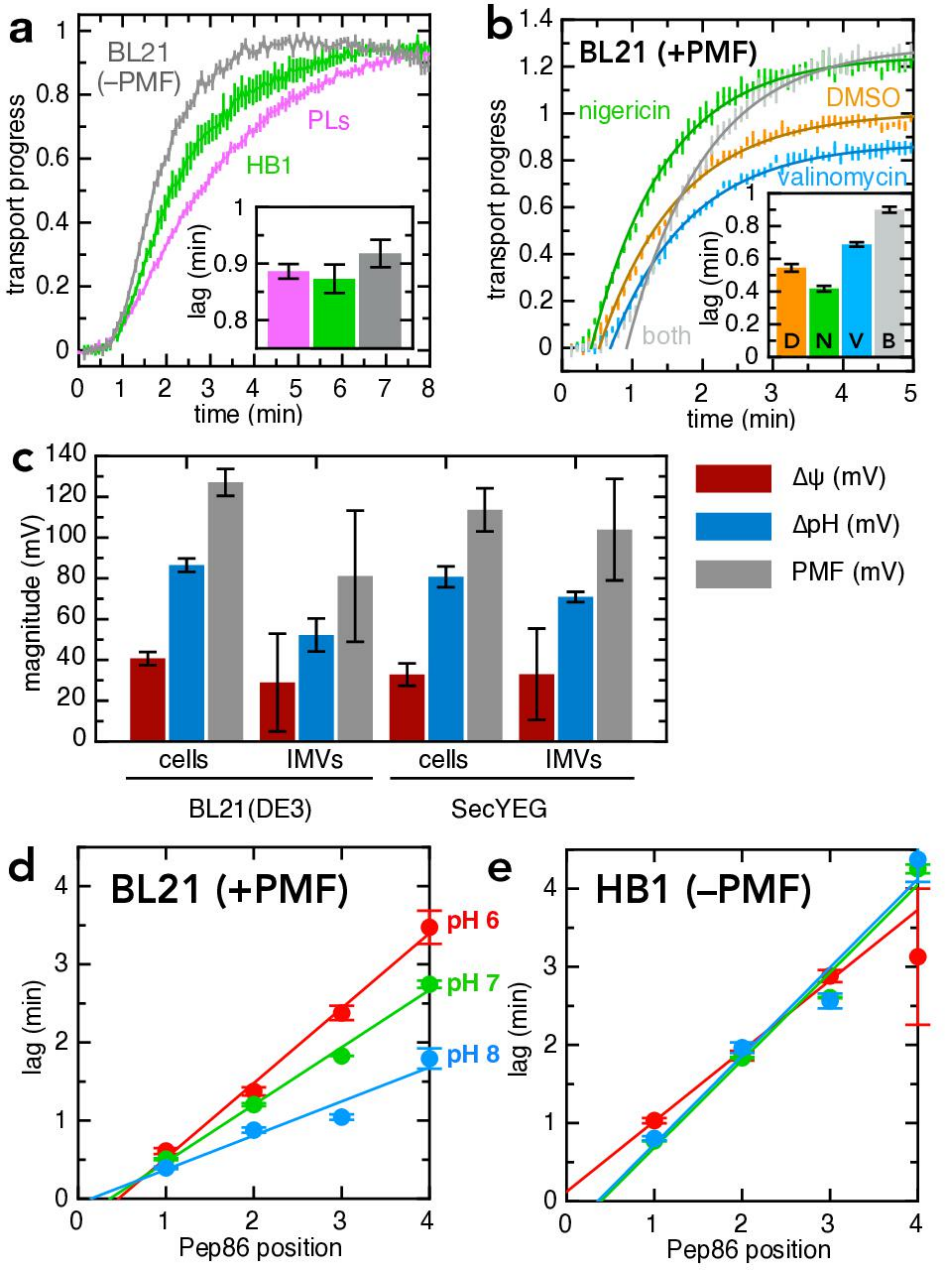
PMF stimulates transport primarily via ∆ψ. (**a**) Transport of pSpy-pep86 into PLs (pink), HB1 IMVs (lacking ATP synthase; green) and BL21 IMVs in the presence of valinomycin and nigericin (grey). Data points and their heights represent the average and SEM from three (PLs), six (HB1) and six (BL21) experimental replicates. Best fit lags (zoomed in for clarity) are shown as an inset; none of the differences in lag are statistically significant as determined by a two-tailed t-test (p = 0.69, 0.31 and 0.20, for PLs-HB1, PLs-BL21, and HB1-BL21, respectively). (**b**) Transport of pSpy-pep86 into BL21 IMVs in the presence of DMSO (orange), valinomycin (blue), nigericin (green), or both ionophores (grey). Data points and their heights represent the average and SEM from six replicates, and lines show the best fit to the single exponential + lag model. Best fit lags are shown inset, and all ionophores have a statistically significant effect on lag as determined by a two-tailed t-test (p = 0.0015, 0.00041 and 3.6 × 10^–7^ for nigericin, valinomycin and both, respectively). (**c**) Comparison of the proton-motive force (PMF, grey) and its two components (∆ψ, red and ∆pH, blue), generated by whole cells and inverted membrane vesicles (IMVs), in BL21(DE3) cells both normal and overexpressing SecYEG. Error bars indicate the standard deviation from three biological replicates (for cells) or three technical replicates (for IMVs). Underlying data and statistics shown in Supplementary file 3d; note that only total PMF in pBAD cells vs IMVs is statistically different (p = 0.0084). (**d**) Lags for import of the pSpy_4x_ series as a function of active pep86 position, into BL21 IMVs at pH 6 (red), 7 (green), and 8 (blue). Data and error bars are the average and SEM from three replicates. (**e**) As in panel (d), but with HB1 IMVs. Figure 6—source data 1.Raw data for Figure 6a–b, and Figure 6—figure supplement 1. Figure 6—source data 2.Raw data for Figure 6c. Figure 6—source data 3.Raw data for Figure 6d. Figure 6—source data 4.Raw data for Figure 6e.

To generate a continuous and stable PMF, we switched from PLs to IMVs purified from cells (over-)producing SecYEG and 11S (see Pereira et al., 2019). IMVs derived from normally functioning *E. coli* strains contain F_1_F_o_-ATP synthase, which works in reverse to produce a PMF upon addition of exogenous ATP. IMVs differ from PLs in that they contain other, native inner membrane proteins, albeit at much lower stoichiometry relative to SecYEG compared to native membranes. We started by comparing transport of pSpy-pep86 into PLs (Figure 6a, pink) with transport into IMVs derived from a cell line lacking F_1_F_o_-ATP synthase, and therefore unable to generate PMF from ATP (HB1; green in Figure 6a). The results show that lag – which corresponds to transport time (see Figure 2b and Allen et al., 2020) – is identical for both, but IMV transport reaches completion faster, meaning a lower probability that transport stalls or fails. Presumably, this enhanced transport processivity is caused by differences between IMVs and PLs – either a specific effect of auxilliary Sec components, or non-specific differences in the membrane environment.

**Figure 6—figure supplement 1.**
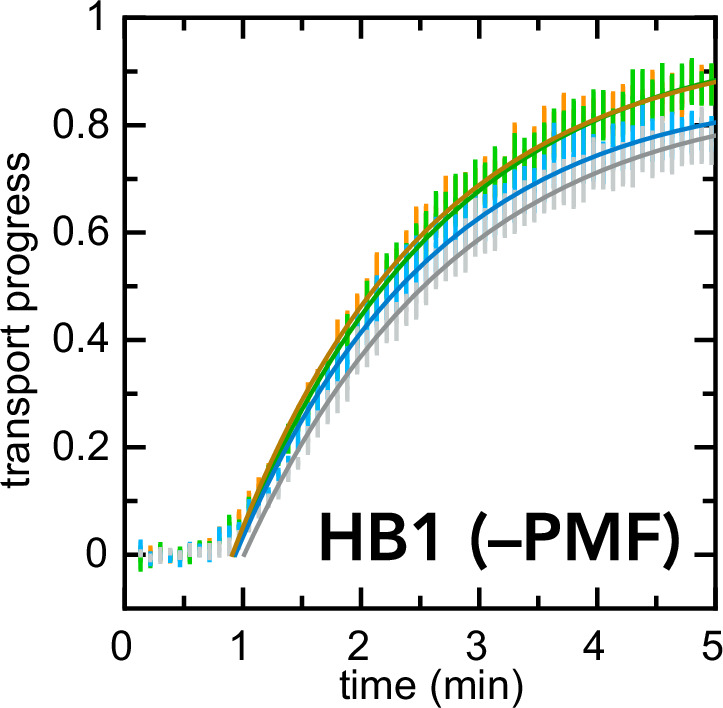
Exactly as in Figure 6b, but with HB1 IMVs instead of BL21 IMVs. In this case, inhibitors have little or no effect on the kinetics of import.

**Figure 6—figure supplement 2.**
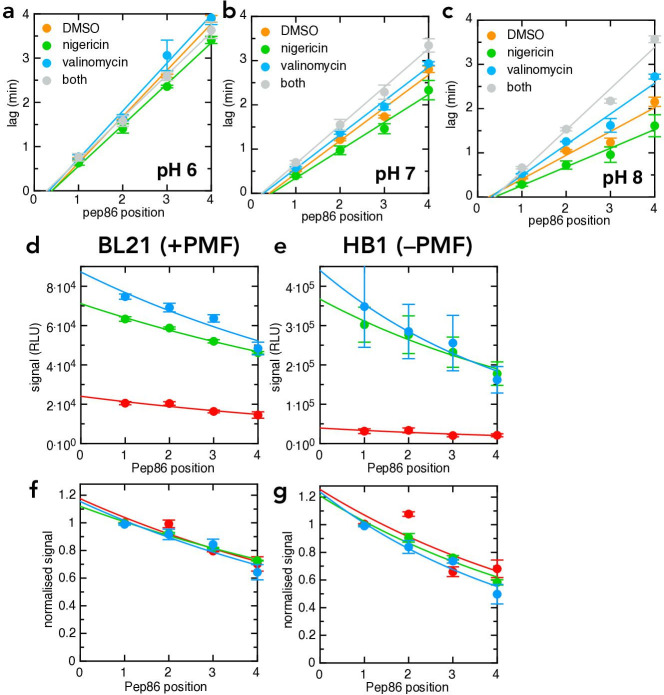
Additional transport data for the pSpy_4x_ series. (**a–c**) Transport lag for the LDDD series in the presence of DMSO (orange), nigericin (green), valinomycin (blue) or nigericin and valinomycin together (grey), at pH 6 (panel **a**), pH 7 (panel **b**) or pH 8 (panel **c**). Data points and error bars are the average and SEM from three experimental replicates (six for DMSO). (**d-g**) Amplitudes from the same primary experiments for which lag was determined in Figure 6c–d. (**d**, **–e**) are unnormalised, and show that pH strongly reduces total signal both in BL21 (**d**) and HB1 (**e**) IMVs. (**f,g**) are normalised to the value for pSpy_LDDD_, and show that the probabilities of pre-protein becoming trapped in the channel (reflecting *k*_block_) are indistinguishable at all three pH values. Figure 6—figure supplement 2—source data 1.Raw data for Figure 6—figure supplement 2a-c.

In order to compare transport with and without PMF under otherwise identical conditions, we used IMVs with functional F_1_F_o_-ATP synthase (BL21) and either added (–PMF) or omitted (+PMF) two ionophores: valinomycin, a potassium ionophore that specifically depletes ∆ψ when K^+^ is present in the buffer; and nigericin, an electroneutral K^+^/H^+^ antiporter that dissipates the ∆pH only under the same conditions. Together, these should eliminate PMF entirely; and indeed, as expected, when both ionophores are present transport into BL21 IMVs give a similar lag to PLs or HB1 IMVs (grey in Figure 6a, see also inset). In the absence of ionophores (i.e. +PMF), transport (lag) is about about twice as fast as in their presence (orange vs grey in Figure 6b). Surprisingly, however, the total amplitude is slightly higher with the ionophores. As import is largely single turnover under the experimental conditions used (one pre-protein per SecYEG), an increase in amplitude suggests either that pre-proteins are less likely to become irrevocably stuck in the channel during transport, or that more of the SecYEG sites are active. Alternatively, the lowered pH inside the PL lumen in the presence of PMF might cause a reduction of NanoLuc signal – either by affecting NanoLuc directly or reducing the accessibility of pep86 – that is reversed by nigericin.

To separate the effects of ∆ψ and ∆pH, we next measured import with each of the two ionophores individually. Valinomycin alone (no ∆ψ, ∆pH increases to compensate) slows transport and produces a small reduction in amplitude (blue in Figure 6b), while nigericin (no ∆pH, increased ∆ψ) appears both to speed up transport and increase the maximum amplitude (green in Figure 6b). These effects are not caused by non-specific effects of the ionophores, as they are not observed for transport into HB1 IMVs (Figure 6—figure supplement 1a). The faster import with nigericin most likely arises from an increase in ∆ψ caused by the dissipation of ∆pH; but this cannot explain the increase in amplitude, which is maintained even in the presence of valinomycin. A reasonable hypothesis is that nigericin is alleviating an effect on signal from the lowered pH in the energised IMV lumen.

While the effects of PMF and the individual ionophores are consistent and reproducible, they are relatively small – certainly not sufficient to bridge the difference in rate between ATP-only driven transport in vitro and the estimated two orders of magnitude faster transport rate in vivo (Allen et al., 2020; Cranford-Smith and Huber, 2018). We therefore measured the magnitude of PMF and its individual components in our purified IMVs, and compared them to the cells from which they are derived (Figure 6c; see Materials and methods for details). The results suggest that the ∆ψ produced by the reverse action of ATP synthase in purified IMVs is comparable to that of intact, respiring *E. coli*, and that overexpression of SecYEG also makes little difference (Figure 6c). However, the absolute measured value of ∆ψ is much lower than is generally reported for *E. coli* (~150 mV; McMillan et al., 2007), and it is also possible that protein transport itself consumes PMF in vitro, as we have observed for mitochondrial protein import (Ford et al., 2021). Therefore, we cannot conclusively determine the extent to which PMF contributes to pre-protein transport in the absence of auxilliary factors.

### Stimulation of transport by PMF is pH-dependent

The IMV lumen volume is only a tiny fraction of the total reaction volume in the transport assay (< 1/5000), so ∆pH presumably manifests as pH decrease inside the vesicle with negligible effect on the bulk pH outside. This contrasts with the situation in a living cell, where ∆pH primarily affects the pH on the cytosolic side of the membrane. Yet if deprotonation of lysines is part of the mechanism of PMF stimulation, then it is the pH experienced by the pre-protein before it is transported that matters – perhaps explaining why nigericin does not slow down transport in vitro.

To test the effect of topologically cytoplasmic pH, we prepared IMVs at three different pHs (6.0, 7.0, and 8.0) and measured import in the same buffer. In the absence of PMF, both the internal and external pH should be the same, and equal to the starting value; generating a PMF will acidify the vesicle lumen but leave the outside pH essentially unaffected. To test the effect of this on transport rate and PMF stimulation we used the pSpy_4x_ series described previously (Allen et al., 2020). This comprises four proteins identical except for the number of copies of mSpy proteins that must pass through SecY before active pep86 becomes available to bind 11S; a plot of lag against active pep86 position therefore gives a straight line with a slope equal to the transport rate in Spy.min^–1^ (Allen et al., 2020).

Just as observed for native pSpy (above), transport is considerably faster (~2 fold) with PMF (BL21 IMVs) than without (HB1 IMVs) at pH 8.0 (Figure 6d vs Figure 6e, blue data). However, as pH decreases, this difference vanishes: at pH 6.0, there is very little PMF effect on rate (Figure 6d–e). The same effect is observed when using ionophores, with the stimulatory effect of nigericin on amplitude also amplified at high pH (Figure 6—figure supplement 2a-c). While this does not prove that it is specifically the pre-protein that is deprotonated, it is evidence that the mechanism of transport stimulation by ∆ψ is dependent on cytosolic pH.

It should also be noted that the overall import signal is much lower at low pH (Figure 6—figure supplement 2d-e). This amplitude reflects the number of functional SecYEG import sites, the probability of transport blocking and the activity of the formed NanoLuc – so a number of things could be causing this reduction. These include faster deterioration of SecYEG during the IMV preparation or lower NanoLuc signal at low pH. Once total amplitude is accounted for, however, the rate at which pSpy_4x_ becomes irreversibly trapped in the channel appears to be largely unaffected by pH (Figure 6—figure supplement 2f-g), ruling this out as a cause.

### PMF stimulation of transport is not measurably dependent on pre-protein charge

**Figure 7.**
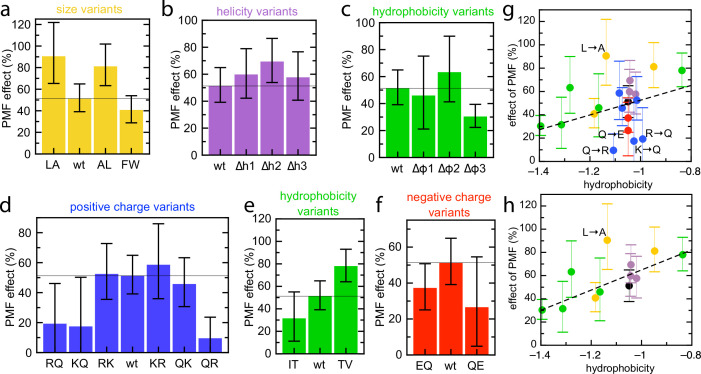
Effect of PMF on transport of the pSpy_XLX_ variants. (**a–f**) Stimulatory effect of PMF on transport of the variable region of all the pSpy_XLX_ variants. These were calculated from the difference in lag for import into BL21 IMVs in the presence and absence of valinomycin and nigericin (see Materials and methods for details and Figure 7—figure supplement 1 for transport times), where 0% is no difference and 100% is a halving of lag. Error bars are calculated by calculating minimum and maximum values from the SEMs from five replicates of each. (**g,h**) PMF effects (coloured as in panels **a–f**) as a function of average hydrophobicity score for the amino acids in each variable mSpy, calculated using the values in Kyte and Doolittle, 1982. (**g**) Entire data set, with best fit line (*r* = 0.271). (**h**) Data excluding the charge variants (i.e. the blue and red values; *r* = 0.681). Outliers are marked directly on the plots. Figure 7—source data 1.Raw data for Figure 7 without ionophores. Figure 7—source data 2.Raw data for Figure 7 with ionophores.

If the PMF primarily is acting on charged residues – promoting transport of negative changes and inhibiting diffusion of arginines – we would expect the magnitude of PMF stimulation to be dependent on the number of charged residues. We therefore measured transport of each of the pSpy_XLX_ variants into BL21 IMVs, both in the presence and absence of valinomycin and nigericin, and calculated the stimulatory effect of PMF on transport time for each (see Materials and methods). The results are shown in Figure 7a–f, with 0% meaning no effect of PMF and 100% a halving of lag in the presence of PMF. As expected, transport is faster in the presence of PMF for all variants. Unexpectedly, however, we see little indication that PMF is acting on charged residues. Indeed, all variants where the number of charges is altered show reduced PMF effect relative to native mSpy – even Q→E, which one might reasonably expect to be assisted by ∆ψ regardless of other factors.

**Figure 7—figure supplement 1.**
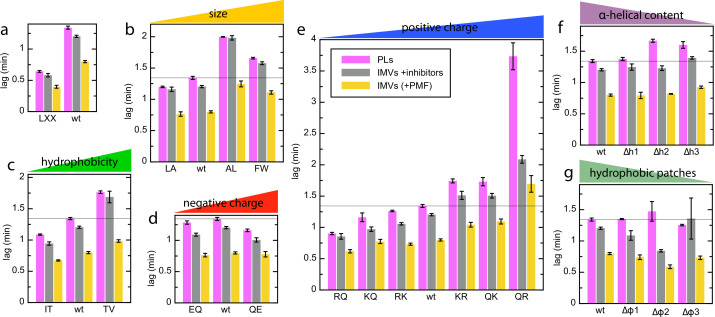
Complete lag data for the pSpy variants in different vesicle types. (**a–g**) Lags for transport of all pSpy_XLX_ variants into PLs (pink; same data as in Figure 2) and BL21 IMVs in the absence (yellow) or presence (grey) of valinomycin and nigericin (raw data for the PMF stimulation data in Figure 7). Data are the average and SEM from three (PLs; 11 for wt) or five (IMVs; 17 and 18 for wt without and with ionophores, respectively) experimental replicates.

The only amino acid property that appears to correlate with magnitude of the PMF effect is hydrophobicity, in that more hydrophobic variants exhibit higher stimulation by PMF. A scatter plot of PMF effect vs hydrophobicity shows this more clearly – a straight line through all the points shows a weak correlation (Figure 7g; *r* = 0.271), which becomes stronger if the charge variants, which mostly show reduced PMF effect, are omitted (Figure 7h; *r* = 0.681). While the large error bars (caused by dividing two experimentally determined numbers) preclude confident assignment of PMF effect to hydrophobic residues, it is the only general amino acid property that has any noticeable effect on this parameter.

Although it is certainly possible that PMF is acting on hydrophobic residues, for example by promoting conformational changes in SecY, the correlation is weak and it would be surprising to us if this were a more significant factor than electrophoresis – particularly given that charged residues in the loops of membrane proteins do experience ∆ψ from the opposite side of the membrane (Ismail et al., 2015). A more plausible explanation for this result is that the PMF effect in the IMV assay is confounded by some other factor. For example, there might be differences in folding behaviour that affect how the pre-protein interacts with the Sec system; a threshold effect of PMF that is not reached in our in vitro system (see Figure 6c); or some contribution by other, unidentified components of IMVs. The exact relationship between PMF, the translocation machinery and pre-protein sequence therefore remains an open question.

## Discussion

The landmark solution of the first atomic structure of SecYEG in complex with SecA, over a decade ago (Zimmer et al., 2008), inspired multiple hypotheses for the underlying mechanism for the transport of pre-proteins across membranes. However, the difficulties obtaining quantitative data on transport itself have, until recently, prevented these from being properly tested. Here, we have applied the newly developed, high time-resolution NanoLuc transport assay (Pereira et al., 2019) to a set of model pre-proteins, designed to capture the rate of transport for amino acid sequences with different properties. Our results reveal a surprisingly simple set of rules for how fast pre-protein is transported through SecYEG: arginine is by far the slowest amino acid to transport, accounting for over 2/3 of the total transport time of the model pre-protein pSpy. Other residues that make a difference are lysines, and bulky residues such as tryptophan, with diffusion of bulky and positive patches probably accounting for the individual steps observed in transport (Figure 1, purple balls).

In our experiments, transport of arginines is slow and rate-limiting. In contrast, lysines behave much more like neutral residues, suggesting that they are at least partially deprotonated before passage across the membrane, a proposition supported by computational analysis of the SecYEG-A structure bound to various model pre-cursors. This deprotonation is presumably facilitated by SecA, with the energy required coming either from ∆pH – protons are more readily removed at the higher pH on the cytosolic side of the membrane – and/or the hydrolytic cycle of ATP, with nucleotide state-induced conformational changes affecting the p*K*_a_ of lysines in the channel. The fact that ∆pH does not appear to have a major effect on transport rate would suggest that the latter is more important, although we interpret these results with caution: the way ∆pH manifests in the assay (lower pH at the channel exit) is not necessarily exactly the same as in vivo (primarily elevated pH at the channel entrance), and our IMV preparations have a low total PMF compared to literature values for *E. coli* cells.

**Figure 8.**
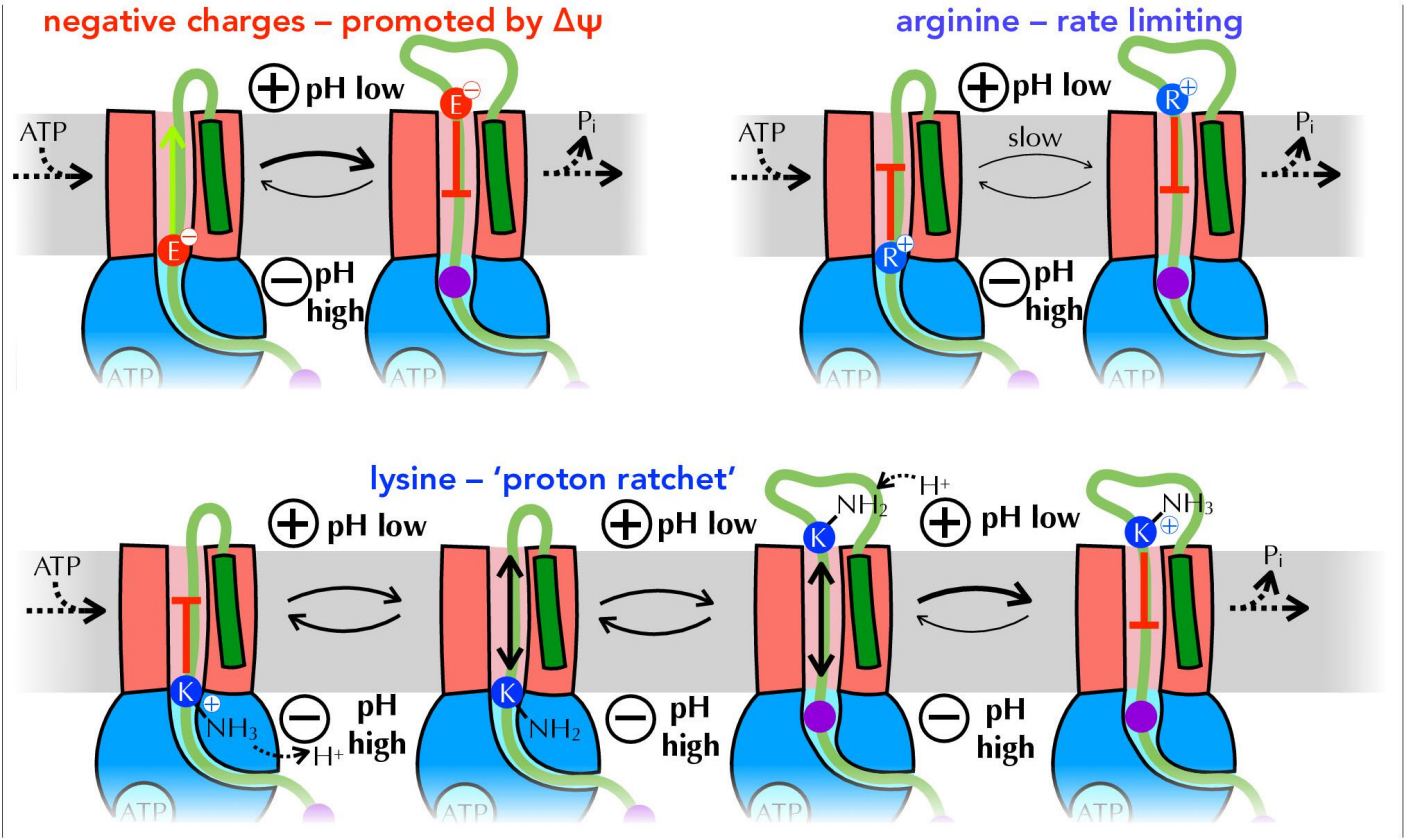
Summary of proposed PMF and ratcheting effects of pre-protein transport. Closeup of step *iii* from Figure 1, where a negative amino acid (red, top left), arginine (blue, top right) or lysine (blue, bottom) is present at the entrance to SecY. ∆ψ is expected to promote the forward diffusion of negative charges, and inhibit their reverse movement. Movement of arginine is unfavourable in either direction, with export especially unfavourable when ∆ψ is present; hence transport of arginine is rate-limiting for the entire process. Lysine is deprotonated at the cytosolic face of SecY (by ∆pH and/or ATP hydrolysis by SecA), allowing its free diffusion across the membrane, and preventing backsliding once it is reprotonated on the other side.

In addition to allowing easy passage of lysine through the SecY channel, which is strongly selective against cations, deprotonation could stimulate transport in two other ways. Firstly, it gives all translocating pre-proteins a net negative charge: as illustrated in Figure 8, diffusion of negative charges (aspartic and glutamic acid) is likely to be promoted by ∆ψ (Ismail et al., 2015), while only arginines (which are under-represented in secreted proteins, see Figure 5) inhibit transport. Additionally, once deprotonated lysines emerge from SecY into the lower pH environment of the periplasm and absent the p*K*_a_ perturbing properties of SecA, they will swiftly be reprotonated and thus unable to diffuse back. This 'proton ratchet' effect will prevent backsliding of transported lysines, and thereby contribute to the forward progression of translocation (Figure 8).

The computational results above were derived from peptides with no other charged residues nearby. Presumably, however, sequence context will have an effect in real life; for example a positive charge adjacent to a negative charge might be harder to deprotonate but easier to transport through the channel, as the two partially cancel each other out. Conversely, a long stretch of consecutive lysines might overwhelm the ability of the Sec machinery to strip and dissipate protons. Poly-lysine is not generally a feature of secreted proteins, but it has previously been used as evidence that positively charged residues are hard to transport (Liang et al., 2012; Nouwen et al., 2009); possibly this strong effect is conferred by the presence of multiple consecutive lysines, as opposed to ones spread evenly through the sequence. The importance of detailed sequence context, along with the compromised PMF in purified IMVs, might also explain the apparently contradictory results we obtain for PMF stimulation of charged pSpy_XLX_ variants.

While arginine has by far the most striking effect of any amino acid, other substitutions also impact the rate of transport. Large amino acids are transported more slowly than smaller ones, most likely because there is more resistance to them diffusing though SecY. Knocking out stretches of α-helix also slows transport, consistent with the Sec system acting on these specifically (Corey et al., 2019). The effect of hydrophobicity, however, is less clear cut: while more hydrophobic sequences are generally transported more slowly, the correlation is not absolute, and knocking out hydrophobic patches has little if any effect on transport. Furthermore, the assay does not allow us to distinguish between general mechanistic effects – for example hydrophobic sequences passing through the channel more slowly – and sequence-specific effects, such as specific hydrogen bonds inhibiting passage of a single part of the pre-protein. Nor are we able to measure the intrinsic structure of the pre-protein before it is transported (Best, 2020), although this is likely to be affected by sequence and in turn affect the rate of diffusion through the channel. Resolving these issues will most likely require studying transport of very short protein sequences at the single molecule level.

In addition to the direct mechanistic insights that can be discerned from the above results, they also have more general implications for the nature of protein secretion. It has previously been shown that a lower propensity for folding and the presence of hydrophobic patches are hallmarks of bacterial secreted proteins (Chatzi et al., 2017); to this we can now add a reduction in the number of arginines. Intriguingly, it also seems that any alterations to the charged residues in Spy – either adding or removing positive or negative charges – reduces the magnitude of PMF stimulation. This perhaps indicates that the sequence of Spy is already well optimised for secretion in terms of charge distribution. These observations all imply that ’secretability' is an important evolutionary constraint on proteins that localise outside the cytosol, particularly very abundant proteins such as Spy (which can account for ~25% of periplasmic upon induction Quan et al., 2011). This will have important ramifications for the rational design of secretion-competent proteins for biotechnology or synthetic biology applications. Our results further suggest that replacing arginines with lysines will be a simple but effective way to achieve higher secretability.

Above all, the identification of steps in the translocation process through SecYEG is important because their properties enable us to distinguish between diffusional (described above) and power-stroke (Catipovic et al., 2019; Catipovic and Rapoport, 2020) mechanisms of translocation. The steps of the translocation reaction we identify could in theory correspond either to average ratchet lengths during diffusion, or to individual piston motions of a power-stroke. But in the former case the steps would vary with the properties of the translocating pre-protein, as indeed they do; steps in a power stroke, meanwhile, would depend on the geometry of SecA and the conformational changes associated with ATP turnover, and thus be invariant. Ratcheting has the further benefit of allowing other factors to contribute to pre-protein transport, including the PMF; pre-protein folding on the outside over the inside (Corey et al., 2019); and the action of auxiliary Sec components such as SecDF (Tsukazaki, 2018) and periplasmic chaperones (Fürst et al., 2018; Knyazev et al., 2018).

The observation that SecA is required to allow positively charged lysines through the SecY channel might plausibly also have implications for topogenesis of membrane proteins. The ‘positive-inside rule’ (Cymer et al., 2015; von Heijne, 1989) – that loops with more positive character are retained in the cytosol – may in part reflect slower kinetics of transporting such loops across the membrane.

## Materials and methods

### Translocation substrate production

To produce the pSpy_XLX_ variants, genes for each variant pSpy were first either synthesised (GeneArt, Thermo Fisher Scientific; pSpy_R→Q_, pSpy_K→Q_, pSpy_R→K_, pSpy_K→R_, pSpy_Q→K_, pSpy_Q→R_, pSpy_E→Q_, pSpy_Q→E_, pSpy_A→L_, pSpy_L→A_, pSpy_I→T_, pSpy_T→V_) or the mutations introduced by site-directed mutagenesis (QuikChange, Agilent; pSpy_∆h1_, pSpy_∆h2_, pSpy_∆h3_, pSpy_∆φ1_, pSpy_∆φ2_, pSpy_∆φ3_, pSpy_F→W_). A gene for pSpy_LX_ was created by removing the dark peptide from pSpy_LD_ (in pBAD/*myc*-His C; Allen et al., 2020) using QuikChange. Each variant mSpy was then cloned between the first mSpy and the pep86 sequences using site directed ligase independent mutagenesis (Chiu et al., 2004). For pSpy_LXX_, the wild type mSpy gene was instead cloned after the pep86 sequence. A complete list of new protein sequences is shown in Supplementary file 1.

All translocation substrates (including the pSpy_4x_ variants; Allen et al., 2020) were expressed as for native pSpy (Pereira et al., 2019), then purified using a new, streamlined protocol. Cell pellets were resuspended 1:7 (v/v) in 7 M guanidine.HCl on ice, incubated for 30 min, then centrifuged at 100,000 g for 30 min. The supernatents were next bound to Ni^+2^ affinity resin (~10 x bead volume) by rotating gently for 30 min at 4 °C, followed by decanting into empty 10 ml gravity flow columns and washing with ≥5 bed volumes tris/urea buffer (20 mM Tris-HCl pH 8.0, 6 M urea) supplemented with 30 mM imidazole. Protein was eluted using tris/urea buffer supplemented with 330 mM imizadole, then passed over a Q-sepharose column to remove nucleic acid contamination. Finally, the purified proteins were concentrated and imidazole removed by spin concentration.

### Production of other translocation reagents

Inverted membrane vesicles (IMVs) overexpressing SecYEG and membrane-tethered 11S (~11S) were produced from previously described cells strains (Pereira et al., 2019). Cells were grown at 37 °C to mid-log phase (OD_600_ ~0.6) in 2xYT supplemented with 100 mg.ml^–1^ amplicillin and 50 mg.ml^–1^ kanamycin, then SecYEG expression was induced with 0.1% (w/v) arabinose. After 2 hr, the cells were cooled to 20 °C then ~11S expression induced overnight (~16 hr) with 1 mM IPTG before harvesting. For purification, a standard protocol was followed (Corey et al., 2018), but using as buffer either M_6_KM (20 mM MES-KOH pH 6.0, 50 mM KCl, 2 mM MgCl_2_), H_7_KM (20 mM HEPES-KOH pH 6.0, 50 mM KCl, 2 mM MgCl_2_) or M_8_KM (20 mM MOPS-KOH pH 8.0, 50 mM KCl, 2 mM MgCl_2_), depending on the desired final pH.

All other transport reaction components were produced as described previously (Pereira et al., 2019). For PLs, this entailed solubilising dried *E. coli* polar lipid (Avanti) in TKM (20 mM Tris-HCl pH 8.0, 50 mM KCl, 2 mM MgCl_2_) with 1% DDM to 4 mg.ml^–1^, then mixing with purified SecYEG at 3.3 µM and 11S at 20 µM. The mix was then extruded to 400 nm and dialysed (12–14 kDa MWCO) overnight in the cold room (4°C–8°C) against TKM, with buffer exchanges after ~2 hr and ~5 hr. PLs were harvested by centrifugation at 100,000 g for 20 min, then washed three times by resuspending in TKM and recentrifuging. The final PL pellets were resuspended to 1:10 weight/volume with TKM.

### Transport assays

Transport assays were performed and analysed exactly as in Allen et al., 2020, with 2 µM final concentration of pre-protein (unless otherwise stated) to ensure saturation. All raw NanoLuc experimental traces are included as Source Data. For IMV experiments, IMVs and the appropriate pH buffer (M_6_KM, H_7_KM or M_8_KM; pH 8 used unless otherwise stated) were substituted for PLs and TKM, but all other reaction conditions were kept identical. Where ionophores were used, they were added from a 100 x stock in DMSO to final concentrations of 1 µM (valinomycin) or 2 µM (nigericin), and the corresponding amount of DMSO was added to comparison experiments.

For Berkeley Madonna analysis of the pSpy_XLX_ variants, the model in Allen et al., 2020 was modified to allow different translocation parameters for the native and variant sequences. A visual representation of this new model is shown in Figure 3—figure supplement 1b; the complete model is shown in Supplementary file 2.

To calculate PMF effect, the import lag for pSpy_LXX_ was subtracted from the lag for each pSpy_XLX_ variant, both in the absence (+PMF) and presence (–PMF) of nigericin and valinomycin, to give transport time just for the variable region. We then calculated PMF effect (in %) as:

$$PMF\;effect\;=100\times \left(\frac{lag_{-PMF}}{lag_{+PMF}}-1\right)$$

Errors in lag were take as the SEM of lag from four technical replicates, where +PMF and –‍PMF were identical other than the addition of ionophores, and error bars for PMF effect were generated by calculating upper and lower bounds from these errors.

### Sequence analysis

Protein properties were calculated over a 9 residue window using ProtScale (https://web.expasy.org/protscale/), using values for hydrophobicity from Kyte and Doolittle, 1982, helical propensity from Deléage and Roux, 1987 and bulkiness from Zimmerman et al., 1968. The same scales were used to plot Figure 2—figure supplement 2a.

### Arginine/Lysine ratio determination

The complete proteomes of *E. coli* (strain K12) and *S. meliloti* (strain 1021) were downloaded from UniProt (The UniProt Consortium, 2017) on 1^st^ May 2018 and sorted according whether they are secreted by Sec or Tat system, or unsecreted. For *E. coli*, which is well annotated by UniProt, this was done using information in the 'Signal Peptide' column. For *S. meliloti*, secreted substrates were selected according to UniProt 'Signal Peptide', then classified as Tat if validated as such in Pickering et al., 2012, or Sec otherwise. *B. halodurans* and *B. subtilis* sequences were downloaded on 13th March 2019 and analysed as for *E. coli*, omitting signal peptides flagged as 'Tat-type'. For secretion substrates, the residues corresponding to the SS were removed prior to analysis. The proportion of positive residues that are lysine (Lys/(Lys + Arg)) was calculated for each protein, then plotted as a histogram for each data set (with sample size n): the number of bins was determined by Sturge’s rule (number of bins = 1 + 3.322 * log(n)), and frequency calculated by dividing the population of that bin by n. Fits to single Gaussian curves were performed using Pro Fit (Quansoft).

### Molecular dynamics of substrate moving through SecY

Systems were built using the coordinates of SecYE and a 14 residue stretch of preprotein (residues 778–791) from PDB 5EUL (Li et al., 2016). The pre-protein was changed into a model peptide, with the sequence AGSGSGSGSGGXGA, where X is the residue of interest (K, R, E, Q, W, L or A). The protein coordinates were built into a POPE:POPG membrane using CHARMM-GUI (Jo et al., 2007; Lee et al., 2016). Proteins were described using the CHARMM36m force field (Best et al., 2012), and waters were TIP3P, with K^+^ and Cl^-^ ions added to 0.15 M. The protein side chains were set to their default protonation states, as predicted using propKa3 (Søndergaard et al., 2011), apart from the Lys^0^ residue where included. Systems were energy minimized using the steepest descents method, and subsequently equilibrated with 1000 kJ.mol^–1^.nm^–2^ positional restraints on protein backbone atoms for 5 ns, and then relaxed using production MD for 15 ns in the NPT ensemble at 310 K with the V-rescale thermostat and semi-isotropic Parrinello-Rahman pressure coupling. Time steps of 2 fs were used.

For each residue, 24 independent steered MD simulations were run for each pre-protein, where the substituted residue was pulled in a z-axis direction (up through the channel) using an umbrella potential moving at a rate of 1 nm.ns^–1^, with a force constant of 1000 kJ.mol^–1^.nm^–2^. For each repeat, the total pulling force (taken as the area under the curve) was recorded.

### Modelling of an engaged pre-protein in the SecA-SecYEG complex for pK_a_ analysis

Initial coordinates for SecA-SecYEG were taken from chains A, C, D, and E of PDB 3DIN (Zimmer et al., 2008), with the ADP-BeF_x_ molecule replaced with ATP (Piggot et al., 2012). Simulations were run over 1 µs in a POPC membrane with explicit waters and Na^+^ and Cl^-^ ions to 0.15 M using the OPLS-AA force field (Jorgensen et al., 1996), see Allen et al., 2016 for full details. Taking a 1 µs snapshot as a fully equilibrated starting model, a region of pre-protein 76 residues long was built in an extended configuration through the SecA-SecYEG complex, as described previously (Corey et al., 2016a). The pre-protein was positioned such that it contacted previously identified crosslinking sites in both SecY and SecA (Corey et al., 2016a; Park et al., 2014). The N-terminal SS was modelled as a helix and sited in the SecY lateral gate a per cryo-EM density (Park et al., 2014).

The SecYEG-SecA-pOA-ATP model was then embedded in a POPC membrane, solvated with explicit waters and Na^+^ and Cl^-^ ions to 0.15 M, and subjected to 1 µs MD simulation. Simulations were carried out as previously described (Allen et al., 2016), using the OPLS-AA force field (Jorgensen et al., 1996), in Gromacs 5.0.4 (Berendsen et al., 1995).

### pK_a_ scanning pipeline

To predict the p*K*_a_ of charged residues as they traverse the SecA-SecYEG complex, we constructed a computational pipeline. For 20 different structural snapshots over the final 500 ns of the SecYEG-SecA-pOA simulations, each of the 76 residues in the pre-protein were substituted to lysine in turn, using Scwrl4 (Krivov et al., 2009), for a total of ca. 1500 snapshots. These were then relaxed for 1 ns using MD, and the p*K*_a_ of the target lysine determined using propKa31 (Søndergaard et al., 2011).

### Construction of 1D free energy profiles

To provide a more detailed view of the energetic cost of transporting protonated lysine, we constructed free energy profiles of protonated and deprotonated lysine residues through the channel, using the Martini 2.2 force field (Marrink et al., 2007; Monticelli et al., 2008). Following conversion to Martini, elastic bonds of 1000 kJ mol^–1^ nm^-2^ were applied between all backbone beads within 1 nm. Electrostatics were described using the reaction field method, with a cut-off of 1.1 nm using a potential shift modifier, and van der Waals interactions were shifted from 0.9 to 1.2 nm. Simulations were run in the NPT ensemble, with V-rescale temperature coupling at 323 K and semi-isotropic Parrinello-Rahman pressure coupling.

We used steered MD to construct a 1D reaction coordinate for an Ala-Lys-Ala peptide through the SecY channel, with the collective variable constructed from the z-distance between each of the backbone beads in the tripeptide and 5 backbone beads forming the SecY pore (Met 75, Ile 78, Ile 183, Val 278 and Ser 401 in *G. thermodentrifinicans numbering*). We performed 200 ns umbrella sampling MD, using a z-axis umbrella force constant of 2000 kJ mol^–1^ nm^–2^, in 0.1 nm windows along this coordinate, with the lysine either protonated or deprotonated. Construction of the 1D free-energy profile was achieved for the last 150 ns of each window using the weighted histogram analysis method, implemented in the gmx wham program (Hub et al., 2010). Convergence was determined through analysis histogram overlap.

### Proton-motive force measurements

The PMF of *E. coli* whole cells was determined using the distribution of [^14^C]benzoate and [^14^C]methyltriphenylphosphonium^+^ as previously described (Rao et al., 2001). Cultures were grown exactly as described for the induction of SecYEG, to allow for a better comparison between whole cells and the IMVs from which they are isolated, and adjusted to OD_600_ = 1.0 using fresh expression media before measurement. The internal volume of these cells was estimated using the partitioning of ^3^H_2_O and [^14^C]PEG-4000 as previously described (Rao et al., 2001). The PMF of IMVs was measured using [^14^C]methylamine and [^14^C]potassium isothiocyanate as previously described (Reenstra et al., 1980), with the following modifications: instead of flow dialysis, a vacuum manifold (Millipore) fitted with 0.45 μm HA MF membrane filters (Millipore) was used as previously described (Gebhard et al., 2006). Assays were performed at a membrane concentration of 1 [mg protein]/mL in buffer (50 mM Tris-HCl pH 7.0, 5 mM MgCl_2_, 100 mM KCl). 1 mM ATP was added and 2 minutes later cold 2 mL 0.1 M LiCl was used to stop the reaction and membranes were harvested by vacuum filtration, with a further wash of 2 mL 0.1 M LiCl. 250 nCi (4.5 μM and 4.2 μM of [^14^C]methylamine and [^14^C]potassium isothiocyanate respectively) was added per experiment. The interval volume of 1.09 μL [mg protein]^–1^ has been previously calculated (Reenstra et al., 1980). 2 mL scintillation fluid (Amersham) was added to all samples in 4 mL scintillation vials and counted as previously described (Gebhard et al., 2006; Rao et al., 2001). Protein concentration was determined using the BCA assay (Thermo) using bovine serum albumin as a standard.

## Data Availability

All raw data generated during this study are included as supplementary files, and annotated with the figure they were used in.

## Additional files

Supplementary file 1. List of pre-protein sequences used. Differences from the native pSpy sequence are highlighted in bold. pSpy_LXX_ is identical to pSpy_XLX_, but with Pep86 and its linker (yellow and pink) moved in front of the variable pSpy (black)

Supplementary file 2. Berkeley Madonna models.

Supplementary file 3. Tables of best fits and p-values.
a: p-values for the difference in lag between each variant and wt.
Calculated using a two-tailed t-test (in Microsoft Excel).
b: List of best fit parameters for all pSpy_XLX_ variants
Values and confidence intervals were estimated by fitting each experimental replicate individually (n = 12 for wt, n = 3 for all the others), and using the mean and SEM of the best fits values.
c: p-values for bioinformatic analysis of arginine/lysine ratios.
Analyses performed using a two-tailed t-test in Microsoft Excel. **d: Comparison of the proton-motive force (PMF) generated by whole cells and inverted membrane vesicles (IMVs**). Error bars indicate the standard deviation from three biological replicates (for cells) or three technical replicates (for IMVs). Raw data underlying the figures. For NanoLuc traces, separate runs are demarcated by alternating bold formatting.

Transparent reporting form
