# Supplementary material for: Rate-limiting transport of positively charged arginine residues through the Sec-machinery is integral to the mechanism of protein secretion
Source: eLife. 2022 Apr 29;11:e77586. doi: 10.7554/eLife.77586 (PMC9110029; doi:10.7554/eLife.77586)
Supplement: Supplementary file 1. — Differences from the native pSpy sequence are highlighted in bold. pSpyLXX is identical to pSpyXLX, but with Pep86 and its linker (yellow and pink) moved in front of the variable pSpy (black) [file elife-77586-supp1.docx]

**Suppementary File 1: List of pre-protein sequences used.**

| **Protein name** | **Sequence*** |
| --- | --- |
| pSpy_XLX_  (wt)^†^ | MRKLTALFVASTLALGAANLAHA*ADTTTAAPADAKPMMHHKGKFGPHQDMMFKDLNLTDAQKQQIREIMKGQRDQMKRPPLEERRAMHDIIASDTFDKVKAEAQIAKMEEQRKANMLAHMETQNKIYNILTPEQKKQFNANFEKRLTERPAAKGKMPATAE*ADTTTAAPADAKPMMHHKGKFGPHQDMMFKDLNLTDAQKQQIREIMKGQRDQMKRPPLEERRAMHDIIASDTFDKVKAEAQIAKMEEQRKANMLAHMETQNKIYNILTPEQKKQFNANFEKRLTERPAAKGKMPATAEGSG*VSGWRLFKKIS*GSG*ADTTTAAPADAKPMMHHKGKFGPHQDMMFKDLNLTDAQKQQIREIMKGQRDQMKRPPLEERRAMHDIIASDTFDKVKAEAQIAKMEEQRKANMLAHMETQNKIYNILTPEQKKQFNANFEKRLTERPAAKGKMPATAE*GSG*ENLYFQG*HHHHHH |
| pSpy_R→Q_ | ADTTTAAPADAKPMMHHKGKFGPHQDMMFKDLNLTDAQKQQI**Q**EIMKGQ**Q**DQMK**Q**PPLEE**QQ**AMHDIIASDTFDKVKAEAQIAKMEEQ**Q**KANMLAHMETQNKIYNILTPEQKKQFNANFEK**Q**LTE**Q**PAAKGKMPATAE |
| pSpy_K→Q_ | ADTTTAAPADAKPMMHH**Q**GKFGPHQDMMF**Q**DLNLTDAQKQQIREIM**Q**GQRDQMKRPPLEERRAMHDIIASDTFD**Q**VKAEAQIA**Q**MEEQRKANMLAHMETQN**Q**IYNILTPEQK**Q**QFNANFEKRLTERPAA**Q**GKMPATAE |
| pSpy_R→K_ | ADTTTAAPADAKPMMHHKGKFGPHQDMMFKDLNLTDAQKQQI**K**EIMKGQKDQMK**K**PPLEEKKAMHDIIASDTFDKVKAEAQIAKMEEQ**K**KANMLAHMETQNKIYNILTPEQK**K**QFNANFEK**K**LTE**K**PAAKGKMPATAE |
| pSpy_K→R_ | ADTTTAAPADAKPMMHHKGKFGPHQDMMF**R**DLNLTDAQKQQIREIM**R**GQRDQMKRPPLEERRAMHDIIASDTFD**R**V**R**AEAQIA**R**MEEQRKANMLAHMETQN**R**IYNILTPEQKRQFNANFE**R**RLTERPAAKGKMPATAE |
| pSpy_Q→K_ | ADTTTAAPADAKPMMHHKGKFGPH**K**DMMFKDLNLTDA**K**KQ**K**IREIMKGQRD**K**MKRPPLEERRAMHDIIASDTFDKVKAEA**K**IAKMEE**K**RKANMLAHMET**K**NKIYNILTPEQKK**K**FNANFEKRLTERPAAKGKMPATAE |
| pSpy_Q→R_ | ADTTTAAPADAKPMMHHKGKFGPH**R**DMMFKDLNLTDA**R**KQ**R**IREIMKGQRD**R**MKRPPLEERRAMHDIIASDTFDKVKAEA**R**IAKMEE**R**RKANMLAHMET**R**NKIYNILTPEQKK**R**FNANFEKRLTERPAAKGKMPATAE |
| pSpy_E→Q_ | ADTTTAAPADAKPMMHHKGKFGPHQDMMFKDLNLTDAQKQQIR**Q**IMKGQRDQMKRPPLE**Q**RRAMHDIIASDTFDKVKA**Q**AQIAKME**Q**QRKANMLAHM**Q**TQNKIYNILTP**Q**QKKQFNANF**Q**KRLT**Q**RPAAKGKMPATAE |
| pSpy_Q→E_ | ADTTTAAPADAKPMMHHKGKFGPH**E**DMMFKDLNLTDA**E**KQ**E**IREIMKGQRD**E**MKRPPLEERRAMHDIIASDTFDKVKAEA**E**IAKMEE**E**RKANMLAHMET**E**NKIYNILTPEQKK**E**FNANFEKRLTERPAAKGKMPATAE |
| pSpy_L→A_ | ADTTTAAPADAKPMMHHKGKFGPHQDMMFKD**A**N**A**TDAQKQQIREIMKGQRDQMKRPP**A**EERRAMHDIIASDTFDKVKAEAQIAKMEEQRKANM**A**AHMETQNKIYNI**A**TPEQKKQFNANFEKR**A**TERPAAKGKMPATAE |
| pSpy_A→L_ | ADTTTAAPADAKPMMHHKGKFGPHQDMMFKDLNLTD**L**QKQQIREIMKGQRDQMKRPPLEERRAMHDII**L**SDTFDKVKAE**L**QIAKMEEQRK**L**NMLAHMETQNKIYNILTPEQKKQFN**L**NFEKRLTERPA**L**KGKMPAT**L**E |
| pSpy_I→T_ | ADTTTAAPADAKPMMHHKGKFGPHQDMMFKDLNLTDAQKQQ**T**RE**T**MKGQRDQMKRPPLEERRAMHD**TT**ASDTFDKVKAEAQ**T**AKMEEQRKANMLAHMETQNK**T**YN**T**LTPEQKKQFNANFEKRLTERPAAKGKMPATAE |
| pSpy_T→V_ | ADTTTAAPADAKPMMHHKGKFGPHQDMMFKDLNL**V**DAQKQQIREIMKGQRDQMKRPPLEERRAMHDIIASD**V**FDKVKAEAQIAKMEEQRKANMLAHME**V**QNKIYNIL**V**PEQKKQFNANFEKRL**V**ERPAAKGKMPA**V**AE |
| pSpy_∆h1_ | ADTTTAAPADAKPMMHHKGKFGPHQDMMFKDLNLTDAQKQQIREIMKGQRDQMKRPP**IDD**RR**GV**HDIIASDTFDKVKAEAQIAKMEEQRKANMLAHMETQNKIYNILTPEQKKQFNANFEKRLTERPAAKGKMPATAE |
| pSpy_∆h2_ | ADTTTAAPADAKPMMHHKGKFGPHQDMMFKDLNLTDAQKQQIREIMKGQRDQMKRPP**IDD**RR**GV**HDIIASDTFDKVK**GDV**QIAKMEEQRKANMLAHMETQNKIYNILTPEQKKQFNANFEKRLTERPAAKGKMPATAE |
| pSpy_∆h3_ | ADTTTAAPADAKPMMHHKGKFGPHQDMMFKDLNLTDAQKQQIREIMKGQRDQMKRPP**IDD**RR**GV**HDIIASDTFDKVK**GDV**QIAKMEEQRKAN**VIG**H**VD**TQNKIYNILTPEQKKQFNANFEKRLTERPAAKGKMPATAE |
| pSpy_∆φ1_ | ADTTTAAPADAKPMMHHKGKFGPHQDMMFKDLNLTDAQKQQIREIMKGQRDQMKRPPLEERRAMHD**QQ**ASDTFDKVKAEAQIAKMEEQRKANMLAHMETQNKIYNILTPEQKKQFNANFEKRLTERPAAKGKMPATAE |
| pSpy_∆φ2_ | ADTTTAAPADAKPMMHHKGKFGPHQDMMFKDLNLTDAQKQQIREIMKGQRDQMKRPPLEERRAMHD**QQ**ASDTFDK**N**KAEAQ**Q**AKMEEQRKANMLAHMETQNKIYNILTPEQKKQFNANFEKRLTERPAAKGKMPATAE |
| pSpy_∆φ3_ | ADTTTAAPADAKPMMHHKGKFGPHQDMMFKDLNLTDAQKQQIREIMKGQRDQMKRPPLEERRAMHD**QQ**ASDTFDK**N**KAEAQ**Q**AKMEEQRKANMLAHMETQNK**Q**YN**Q**LTPEQKKQFNANFEKRLTERPAAKGKMPATAE |
| pSpy_F→W_ | ADTTTAAPADAKPMMHHKGK**W**GPHQDMM**W**KDLNLTDAQKQQIREIMKGQRDQMKRPPLEERRAMHDIIASDT**W**DKVKAEAQIAKMEEQRKANMLAHMETQNKIYNILTPEQKKQ**W**NAN**W**EKRLTERPAAKGKMPATAE |

*for XLX variants, only the middle (altered) sequence is shown.

^†^colour scheme: SS, *constant mSpy*, variable mSpy, linker, *Pep86*, *TEV site* and his tag.
