# Supplementary material for: Rate-limiting transport of positively charged arginine residues through the Sec-machinery is integral to the mechanism of protein secretion
Source: eLife. 2022 Apr 29;11:e77586. doi: 10.7554/eLife.77586 (PMC9110029; doi:10.7554/eLife.77586)
Supplement: Supplementary file 2. [file elife-77586-supp2.docx]

**Supplementary File 2**

**Model 1: Berkeley Madonna model for XLX variants (n_var_ >= 3)**

METHOD RK4

STARTTIME = 0

STOPTIME = 10

DT = 0.001

{*** Initial parameters ***}

{rate constants (in µM and min, as appropriate)}

k_on = 0.96

k_off = 0.085

k_step_wt = 4.27359

k_init = k_step_wt

k_block_wt = 0.31

k_fail_wt = 0.24189

k_step_var = 5

k_block_var = 0.1

k_fail_var = 0.1

{supplied concentrations (µM)}

Lg = 0.005 {11S concentration (final)}

A0 = 0.004 {starting SecYEG concentration}

B0 = 1 {starting pre-protein concentration}

{additional parameters}

n_wt = 5

n_var = 5

brightness = 471.082 {how much signal is produced per nLuc}

{*** Reaction setup ***}

{initiate concentrations}

b_quad = A0 + B0 + (k_off / k_on)

INIT C = (b_quad - SQRT((b_quad * b_quad) - (4 * A0 * B0))) / 2

INIT A = A0 - C

INIT B = B0 - C

INIT D[0..n_wt] = 0

INIT E[1..n_var] = 0

{*** Differential equations ***}

d/dt (A) = (C * k_off) - (A * B * k_on) + (ARRAYSUM(D[*]) * k_fail_wt) + ((ARRAYSUM(E[*]) - E[n_var]) * k_fail_var)

d/dt (B) = (C * k_off) - (A * B * k_on) + (ARRAYSUM(D[*]) * k_fail_wt) + ((ARRAYSUM(E[*]) - E[n_var]) * k_fail_var)

d/dt (C) = (A * B * k_on) - (C * k_off) - (C * k_init)

d/dt (D[0]) = (C * k_init) - (D[0] * k_step_wt) - (D[0] * k_block_wt) - (D[0] * k_fail_wt)

d/dt (D[1..n_wt-1]) = (D[i-1] * k_step_wt) - (D[i] * k_step_wt) - (D[i] * k_block_wt) - (D[i] * k_fail_wt)

d/dt (D[n_wt]) = (D[n_wt-1] * k_step_wt) - (D[n_wt] * k_step_var) - (D[n_wt] * k_block_var) - (D[n_wt] * k_fail_var)

d/dt (E[1]) = (D[n_wt] * k_step_var) - (E[1] * k_step_var) - (E[1] * k_block_var) - (E[1] * k_fail_var)

d/dt (E[2..n_var-1]) = (E[i-1] * k_step_var) - (E[i] * k_step_var) - (E[i] * k_block_var) - (E[i] * k_fail_var)

d/dt (E[n_var]) = (E[n_var-1] * k_step_var)

{*** Output ***}

signal = min(E[n_var], Lg) * brightness

**Model 2: Berkeley Madonna model for XLX variants (n_var_ = 2)**

METHOD RK4

STARTTIME = 0

STOPTIME = 10

DT = 0.001

{*** Initial parameters ***}

{rate constants (in µM and min, as appropriate)}

k_on = 0.96

k_off = 0.085

k_step_wt = 4.27359

k_init = k_step_wt

k_block_wt = 0.31

k_fail_wt = 0.24189

k_step_var = 5

k_block_var = 0.1

k_fail_var = 0.1

{supplied concentrations (µM)}

Lg = 0.005 {11S concentration (final)}

A0 = 0.004 {starting SecYEG concentration}

B0 = 1 {starting pre-protein concentration}

{additional parameters}

n_wt = 5

n_var = 2

brightness = 471.082 {how much signal is produced per nLuc}

{*** Reaction setup ***}

{initiate concentrations}

b_quad = A0 + B0 + (k_off / k_on)

INIT C = (b_quad - SQRT((b_quad * b_quad) - (4 * A0 * B0))) / 2

INIT A = A0 - C

INIT B = B0 - C

INIT D[0..n_wt] = 0

INIT E[1..n_var] = 0

{*** Differential equations ***}

d/dt (A) = (C * k_off) - (A * B * k_on) + (ARRAYSUM(D[*]) * k_fail_wt) + ((ARRAYSUM(E[*]) - E[n_var]) * k_fail_var)

d/dt (B) = (C * k_off) - (A * B * k_on) + (ARRAYSUM(D[*]) * k_fail_wt) + ((ARRAYSUM(E[*]) - E[n_var]) * k_fail_var)

d/dt (C) = (A * B * k_on) - (C * k_off) - (C * k_init)

d/dt (D[0]) = (C * k_init) - (D[0] * k_step_wt) - (D[0] * k_block_wt) - (D[0] * k_fail_wt)

d/dt (D[1..n_wt-1]) = (D[i-1] * k_step_wt) - (D[i] * k_step_wt) - (D[i] * k_block_wt) - (D[i] * k_fail_wt)

d/dt (D[n_wt]) = (D[n_wt-1] * k_step_wt) - (D[n_wt] * k_step_var) - (D[n_wt] * k_block_var) - (D[n_wt] * k_fail_var)

d/dt (E[1]) = (D[n_wt] * k_step_var) - (E[1] * k_step_var) - (E[1] * k_block_var) - (E[1] * k_fail_var)

d/dt (E[2]) = (E[1] * k_step_var)

{*** Output ***}

signal = min(E[2], Lg) * brightness
