# Supplementary material for: Rate-limiting transport of positively charged arginine residues through the Sec-machinery is integral to the mechanism of protein secretion
Source: eLife. 2022 Apr 29;11:e77586. doi: 10.7554/eLife.77586 (PMC9110029; doi:10.7554/eLife.77586)
Supplement: Supplementary file 3. — a: p-values for the difference in lag between each variant and wt. Calculated using a two-tailed t-test (in Microsoft Excel). b: List of best fit parameters for all pSpyXLX variants Values and confidence intervals were estimated by fitting each experimental replicate individually (n = 12 for wt, n = 3 for all the others), and using the mean and SEM of the best fits values. c: p-values for bioinformatic analysis of arginine/lysine ratios. Analyses performed using a two-tailed t-test in Microsoft Excel. d: Comparison of the proton-motive force (PMF) generated by whole cells and inverted membrane vesicles (IMVs). Error bars indicate the standard deviation from three biological replicates (for cells) or three technical replicates (for IMVs). Raw data underlying the figures. For NanoLuc traces, separate runs are demarcated by alternating bold formatting. [file elife-77586-supp3.docx]

**Supplementary File 3: Fit parameters**

**a: p-values for the difference in lag between each variant and wt.**

| AL | 1.16168E-08 |
| --- | --- |
| EQ | 0.265292515 |
| FW | 2.89942E-05 |
| ∆h1 | 0.488388125 |
| ∆h2 | 2.55424E-05 |
| ∆h3 | 0.00045834 |
| IT | 0.000186505 |
| KQ | 0.00870247 |
| KR | 3.87805E-06 |
| LA | 0.011269789 |
| LXX | 6.15403E-09 |
| ∆p1 | 0.8765962 |
| ∆p2 | 0.158087507 |
| ∆p3 | 0.093423493 |
| QE | 0.002919792 |
| QK | 1.78073E-05 |
| QR | 5.28042E-11 |
| RK | 0.124575186 |
| RQ | 1.08241E-06 |
| TV | 1.52642E-06 |

**b: List of best fit parameters for all pSpy_XLX_ variants**

| **variant** | **n** | **k_step,var_** | **k_block,var_** | **k_fail,var_** |
| --- | --- | --- | --- | --- |
| wt | 5.91670 ± 0.19301 | 6.73570 ± 0.24265 | 0.31 (fixed) | 0.06886 ± 0.01291 |
| LA | 4 | 5.07013 ± 0.09586 | 0.31396 ± 0.01771 | 0.24708 ± 0.01415 |
| AL | 5.33333 ± 0.33333 | 2.73244 ± 0.21073 | 0.19021 ± 0.00790 | 0.09855 ± 0.00670 |
| FW | 4 | 2.49783 ± 0.01229 | 0.32322 ± 0.00468 | 0.10616 ± 0.00594 |
| IT | 7 ± 0.57735 | 12.27283 ± 1.05754 | 0.26227 ± 0.02595 | 0.33184 ± 0.04372 |
| TV | 5 | 3.27936 ± 0.05723 | 0.22540 ± 0.00883 | 0.02220 ± 0.00897 |
| EQ | 5.66667 ± 0.33333 | 6.93964 ± 0.83979 | 0.26707 ± 0.02426 | 0.15690 ± 0.02525 |
| QE | 4.66667 ± 0.33333 | 5.66993 ± 0.70510 | 0.19629 ± 0.02956 | 0.53530 ± 0.04485 |
| RQ | ≥20 | ≥64 | 0.25762 ± 0.01797 | 0.39651 ± 0.02940 |
| KQ | 6.66667 ± 0.66667 | 11.35220 ± 2.99614 | 0.28326 ± 0.02741 | 0.11947 ± 0.04241 |
| RK | 6.33333 ± 0.33333 | 8.11439 ± 0.52564 | 0.29334 ± 0.02818 | 0.14943 ± 0.06589 |
| KR | 5 | 3.28732 ± 0.12557 | 0.20297 ± 0.01269 | 0.06205 ± 0.01115 |
| QK | 4.33333 ± 0.33333 | 2.76085 ± 0.24414 | 0.21420 ± 0.00654 | 0.04711 ± 0.02008 |
| QR | 5 | 0.93044 ± 0.06346 | 0.13110 ± 0.00816 | 0.05710 ± 0.01269 |
| ∆h1 | 6 | 6.48068 ± 0.21797 | 0.29663 ± 0.01000 | 0.07904 ± 0.00449 |
| ∆h2 | 7 | 5.32258 ± 0.08994 | 0.22629 ± 0.02141 | 0.07786 ± 0.02570 |
| ∆h3 | 6 | 4.76603 ± 0.29008 | 0.19169 ± 0.00957 | 0.11433 ± 0.02188 |
| ∆φ1 | 4.33333 ± 0.33333 | 4.32668 ± 0.34422 | 0.18862 ± 0.01671 | 0.21557 ± 0.00606 |
| ∆φ2 | 6 ± 0.57735 | 6.13496 ± 1.20729 | 0.21340 ± 0.01078 | 0.27345 ± 0.03692 |
| ∆φ3 | 6 ± 0.57735 | 7.39306 ± 1.17548 | 0.21314 ± 0.02301 | 0.26077 ± 0.05402 |

**c: p-values for bioinformatic analysis of arginine/lysine ratios**

|  | Sec vs unsecreted | Sec vs Tat | Tat vs unsecreted |
| --- | --- | --- | --- |
| *E. coli* | 1.68728E-47 | 0.071764886 | 0.107526671 |
| *S. meliloti* | 3.444E-143 | 0.009989205 | 1.90911E-24 |
| *B. halodurans* | 0.000433917 | – | – |
| *B. subtilis* | 2.44192E-50 | – | – |

**d: Comparison of the proton-motive force (PMF) generated by whole cells and inverted membrane vesicles (IMVs)**

| **Sample** | **Δψ (mV)** | **ZΔpH (mV)** | **PMF (mV)** |
| --- | --- | --- | --- |
| **pBAD cells** | 40.7 ± 3.2 | 86.5 ± 3.3 | 127.1 ± 6.6 |
| **pBAD IMVs** | 28.9 ± 24.0 | 52.2 ± 8.1 | 81.1 ± 32.2^a^ |
| **pSEC cells** | 32.8 ± 5.5 | 80.8 ± 5.1 | 113.6 ± 10.6 |
| **pSEC IMVs** | 33.0 ± 22.4 | 70.9 ± 2.5 | 103.9 ± 24.9 |

^a^Statistically significant vs pBAD cells (*p* = 0.0084, two-way ANOVA, Tukey’s multiple comparison test, 95% C.I.)
